# CCR2 signaling regulates anti-chlamydia T cell immune responses in the airway

**DOI:** 10.1371/journal.ppat.1012912

**Published:** 2025-02-04

**Authors:** Shuaini Yang, Jinxi Yu, Xue Dong, Jiajia Zeng, Lu Tan, Hong Zhang, Ruoyuan Sun, Yuqing Tuo, Jing Yang, Chunxiao Wan, Hong Bai

**Affiliations:** 1 Key Laboratory of Immune Microenvironment and Disease, Tianjin Institute of Immunology, Department of Immunology, School of Basic Medical Sciences, Tianjin Key Laboratory of Cellular and Molecular Immunology, Tianjin Medical University, Tianjin, China; 2 Department of Physical and Rehabilitation Medicine, Tianjin medical University General Hospital, Tianjin, China; 3 Tianjin NanKai Hospital, Tianjin Medical University, Tianjin Key Laboratory of Acute Abdomen Disease Associated Organ Injury and ITCWM Repair, Institute of Integrative Medicine for Acute Abdominal Diseases, Tianjin, China; University of Texas Health Science Center at San Antonio, UNITED STATES OF AMERICA

## Abstract

CCR2, a member of the G protein-coupled receptor (GPCR) superfamily, is widely expressed on monocytes, macrophages, activated T cells, and other cell types, and plays a critical role in coordinating the immune response to various infections. Here we demonstrate that CCR2 expression is significantly elevated during Chlamydia muridarum (*C*. *muridarum*) respiratory infection, and its absence leads to exacerbated susceptibility, as evidenced by significant weight loss, higher bacterial loads, severe lung pathology, and elevated levels of inflammatory cytokines (*il-1β*, *tnfα*, and *il-6*). The absence of *ccr2* impairs both myeloid cell infiltration and T cell responses, which are crucial for effective immune defense. Specifically, *ccr2* deficiency disrupts the differentiation and response of Th1 cells, which are the primary effector lineage responsible for clearing chlamydia through secretion of interferon-gamma (IFN-γ). As a result, there is a significant decrease in CD3^+^CD4^+^IFN-γ^+^ T cells in the lung and spleen, accompanied by reduced levels of IFN-γ protein and mRNA, as well as downregulated mRNA expression of Th1-promoting cytokines (*il-12p35*, *il-12p40*) and transcription factors (*stat4*, *T-bet*), which play crucial roles in Th1 differentiation. Moreover, *ccr2* deficiency greatly diminishes STAT1 phosphorylation, a key regulator of IFN-γ secretion by Th1 cells. Meanwhile, we also observed a significant reduction in IFN-γ secretion by CD8^+^ T cells following *ccr2* deficiency. Conversely, *ccr2*^-/-^ mice exhibit an exaggerated Th2-type immune response, with elevated levels of Th2-promoting cytokines (IL-4), transcription factors (STAT6 and *gata3*), and *il-5*, which together lead to more severe lung tissue damage and increased susceptibility to infection. Furthermore, these mice show higher levels of IL-17 along with an enhanced Th17-type immune response, characterized by increased Th17-promoting cytokines *TGFB*, transcription factors *stat3* and *RORγt*, and *il-21*, suggesting a compensatory mechanism that drives neutrophil infiltration to exacerbate lung inflammation. These findings underscore the pivotal role of CCR2, a chemokine receptor, in orchestrating the immune response to Chlamydia infection by facilitating Th1 cells differentiation while restraining Th2-type and Th17-type immune responses, thereby alleviating pulmonary inflammation.

## Author summary

Chlamydia species are obligate intracellular bacterial pathogens responsible for a wide array of infections, including sexually transmitted diseases and respiratory illnesses. Due to their unique life cycle, Chlamydia infections are challenging to eradicate, and current treatments do not ensure complete clearance, often resulting in chronic or recurrent infections. The mechanisms that regulate host protective immunity against Chlamydia remain poorly understood. In this study, we employed *ccr2*^-/-^ mice to investigate the role of CCR2 in a *Chlamydia muridarum* respiratory infection model. Our findings indicate that CCR2 plays an important role in regulating the immune response during infection. Mice deficient in CCR2 exhibited exacerbated outcomes, including significant weight loss, increased bacterial burdens, and severe pulmonary damage. This was attributed to impaired immune responses, particularly in CD4^+^ T cells, which are critical for combating infections. In the absence of CCR2, mice exhibited impaired Th1 cell differentiation and diminished IFN-γ secretion, both of which are essential for effective pathogen clearance. In contrast, they developed an exaggerated Th2 and Th17 immune response, resulting in heightened inflammation and tissue damage. These findings underscore the role of CCR2 in balancing immune responses, thereby promoting a more effective defense while preventing excessive inflammation, and provide valuable insights into potential therapeutic targets for Chlamydia infections.

## Introduction

Chlamydia species are obligate intracellular bacterial pathogens that rely on host-derived nutrients to support their growth, development, reproduction, and successful infection. They also modulate the metabolic mechanisms and physiological processes of host cells. Three Chlamydia species are pathogenic to human: Chlamydia trachomatis (*C*. *trachomatis*), the leading bacterial cause of sexually transmitted infections and trachoma. Chlamydia psittaci (*C*. *psittaci*), which primarily infects psittacine birds but can be transmitted to humans, causing psittacosis. Chlamydia pneumoniae (*C*. *pneumoniae*), a major causative agent of respiratory tract diseases, including community-acquired atypical pneumonia, bronchitis, pharyngitis, and sinusitis [1,2]. Chlamydia muridarum (*C*. *muridarum*) strain used in this study was originally isolated from infected rodents, such as mice or hamsters. It is commonly employed in basic research on Chlamydia respiratory and genital infections due to its ability to replicate pathological conditions observed in animal models that resemble those in humans [3]. Importantly, this strain specifically infects rodents and poses no risk of infection to humans or other species [4]. Despite advancements in our understanding of Chlamydia biology, the prevention and treatment of these infections remain significant challenges, emphasizing the need for further investigation into their pathogenic mechanisms.

Chemokines, classified into CXC, CC, CX3C, and C subfamilies based on N-terminal cysteine residues [1,5], regulate inflammation by binding to G-protein-coupled receptors, controlling leukocyte adhesion, migration, activation, and balancing Th1/Th2 responses, making them potential therapeutic targets in inflammatory diseases [5,6]. Chemokine receptor 2 (CCR2), expressed on immature dendritic cells (DCs), macrophages, monocytes, neutrophils, and activated T cells [7], guides monocyte migration from the bone marrow to infection sites [8], playing a crucial role in immune defense and the pathogenesis of inflammatory disorders [9]. In *Listeria monocytogenes* infection, the absence of CCR2 leads to a lack of TNF/iNOS-producing (Tip) dendritic cells, resulting in severe TNF and iNOS deficiencies and impaired bacterial clearance [10]. In *Orientia* infection, *ccr2*^-/-^ mice exhibit delayed disease onset, impaired bacterial clearance in both lungs and liver, slower macrophage infiltration, and delayed inflammatory responses [11]. In *Mycobacterium tuberculosis* infection, *ccr2*^-/-^ mice show impaired recruitment of macrophages, DCs, and T cells, delayed T cell priming, and higher bacterial loads in the lungs [12]. In *L*. *neumophila* infection, reduced monocyte migration from the bone marrow to infection sites in *ccr2*^-/-^ mice results in fewer monocyte-derived DCs in the lungs, enhanced pathogen replication, and aggravated disease [8]. In *Yersinia pseudotuberculosis* infection, CCR2-expressing inflammatory DCs are crucial for generating a large and protective YopE69-77-specific CD8^+^ T cell response by translocating YopE into host cell cytosol [13]. Conversely, some studies have shown that lack of CCR2 protects against liver injury [14] and experimental autoimmune encephalitis [15]. However, little is known about its role in chlamydial lung infections.

It is well known that immunity to *C*. *trachomatis* involves many cell types, but CD4^+^ T cells play a key role in protecting the host during natural infection. Specifically, IFN-γ production by CD4^+^ T cells is the primary effector responsible for bacterial clearance [16]. Insufficient IFN-γ, as observed during chronic *C*. *trachomatis* infection, leads to incomplete bacterial clearance and poor immune activation [17]. Chemokines such as RANTES, IFN-γ-inducible protein-10 (IP-10), and macrophage inflammatory protein-1α (MIP-1α), along with their receptor CCR1, are elevated in the lungs during *C*. *muridarum* infections, and this elevation is closely associated with Th1 cell responses, particularly linking MIP-1α to the modulation of these immune responses [18]. The CCL2-CCR2 system plays a crucial role in regulating T cell differentiation by affecting both antigen-presenting cell trafficking and cytokine secretion, as well as exerting effects on differentiating T cells [19]. We speculate that CCR2 may influence the direction of T cell differentiation to modulate host resistance to Chlamydia respiratory infection and lung inflammatory damage.

This study demonstrates the critical role of CCR2 in orchestrating immune responses during Chlamydia lung infections by modulating CD4^+^ T cell subsets, which, in turn, affects the progression and resolution of pulmonary inflammation. These findings suggest that targeting CCR2 signaling could enhance immune responses against respiratory pathogens. Understanding the balance between chemokine receptors and T cell responses can guide the development of new treatments for infectious diseases.

## Materials and methods

### Ethics statement

The ethical guidelines for animal experiments approved by the Committee on the Ethics of Animal Experiments at Tianjin Medical University (permit number SYXK (Tianjin) 2023–0004).

### Mice

In this study, female mice aged 6–8 weeks and weighing 18-22g were selected. C57BL/6 mice (wild-type, WT) were purchased from SiPeiFu Biotechnology (Beijing, China). *Ccr2*^-/-^ mice on C57BL/6 background were initially provided by Dr. Jing Yang (Tianjin Nankai Hospital, Tianjin, China) and bred in the Tianjin Medical University Experimental Animal Center. All mice were housed under specific-pathogen-free conditions in compliance with the ethical guidelines for animal experiments.

### *C*.*muridarum* respiratory tract infection mouse models

Chlamydia muridarum (*C*. *muridarum*), gifted by Professor Xi Yang from the University of Manitoba (Canada), was cultured, purified, and enumerated as previously described [20,21]. The mice (WT and *ccr2*^-/-^) were anesthetized using isoflurane and intranasally inhaled 1×10^3^ inclusion-forming units (IFUs) of *C*. *muridarum* in a 40μL sucrose phosphate glutamic acid (SPG) buffer, establishing respiratory tract infection models for *C*. *muridarum*. As a control, the uninfected mice (0d) received intranasal inhalation of 40μL SPG buffer. These procedures were conducted within a biosafety level 1 (BSL-1) laboratory.

### Quantitative real-time PCR analysis (qPCR)

Sterile isolation of lung tissues from WT and *ccr2*^-/-^ mice infected at different time points was performed, followed by extraction of total RNA using Trizol reagent (Ambion, Austin, Texas, USA). Subsequently, cDNA synthesis was performed using the TransScript One-Step gDNA Removal and cDNA Synthesis SuperMix (TransGen Biotech, Beijing, China) according to the manufacturer’s instructions. The qPCR analysis was conducted on Light Cycler 96 (Roche, Basel, Switzerland) using 2× RealStar Green Fast Mixture (GenStar, Beijing, China). The mRNA expression levels of the target genes were determined using relative quantification. β-actin served as an endogenous reference and the relative expression was calculated using the 2^−ΔΔCt^ method. The primers used in this study were obtained from Shanghai Sangon Biotech, and their sequences are provided in Table 1.

**Table 1:** qPCR primer sequences.

Gene name	Forward Sequence (5’-3’)	Reverse Sequence (5’-3’)
β-actin	GGCTGTATTCCCCTCCATCG	CCAGTTGGTAACAATGCCATGT
ccr2	ATCCACGGCATACTATCAACATC	AAGGCTCACCATCATCGTAG
ccl2	TAAAAACCTGGATCGGAACCAAA	GCATTAGCTTCAGATTTACGGGT
16S rRNA	CGCCTGAGGAGTACACTCGC	CCAACACCTCACGGCACGAG
il-1β	GAAATGCCACCTTTTGACAGTG	TGGATGCTCTCATCAGGACAG
tnfα	CTGAACTTCGGGGTGATCGG	GGCTTGTCACTCGAATTTTGAGA
il-6	TGAACAACGATGATGCACTTGCAG	TAGCCACTCCTTCTGTGACTCCAG
ifn-g	CAGCAACAGCAAGGCGAAAAAGG	TTTCCGCTTCCTGAGGCTGGAT
il-12p35	CAATCACGCTACCTCCTCTTTT	CAGCAGTGCAGGAATAATGTTTC
il-12p40	TGGTTTGCCATCGTTTTGCTG	ACAGGTGAGGTTCACTGTTTCT
stat4	GCACTCAGTAAGATGACGCAG	CCAGTAGGGTAAAGCAGTTCTG
T-bet	AACCGCTTATATGTCCACCCA	CTTGTTGTTGGTGAGCTTTAGC
il-4	GGTCTCAACCCCCAGCTAGT	GCCGATGATCTCTCTCAAGTGAT
il-5	GATGAGGCTTCCTGTCCCTACT	TGACAGGTTTTGGAATAGCATTTCC
gata3	AAGCTCAGTATCCGCTGACG	GTTTCCGTAGTAGGACGGGAC
il-17	TCAGCGTGTCCAAACACTGAG	CGCCAAGGGAGTTAAAGACTT
RORγt	GAGATGCTGTCAAGTTTGGC	TGTAAGTGTGTCTGCTCCGC
TGFB	AAAACAGGGGCAGTTACTACAAC	TGGCAGATATAGACCATCAGCA
il-21	TCATCATTGACCTCGTGGCCC	ATCGTACTTCTTACATTGCAATCCC
stat3	CACCTTGGATTGAGAGTCAAGAC	AGGAATCGGCTATATTGCTGGT

### Lung Chlamydia loads (IFUs detection)

The aseptically separated lung tissues were ground on ice in SPG to prepare a homogenate. After centrifugation at 3000 rpm at 4°C for 30 minutes, the diluted supernatant was added to a confluent monolayer of Hela cells and incubated at 37°C for 2 hours. Then, the DMEM complete medium was replaced. Following cultivation for 40 to 48 hours, the cells were fixed with methanol for 10 minutes. Mouse anti-chlamydia LPS IgG (Invitrogen, Carlsbad, CA, USA) was incubated for 70 minutes, followed by HRP-conjugated goat anti-mouse IgG (Solarbio Beijing China) for an additional 50 minutes. Chlamydia inclusion bodies were visualized using substrate (4-chloro-1-naphthol, Solarbio) and counted under an optical microscope (200× magnification) to assess chlamydia loads in the lung.

### Histopathological analysis and semi-quantitative scoring

Lung tissues from uninfected and *C*. *muridarum*-infected mice were fixed in 4% paraformaldehyde (PFA) (Life-iLab, Shanghai, China) for 48 hours. After fixation, the tissues underwent dehydration, transparency treatment, wax embedding, paraffin sectioning (5 μm), and staining with hematoxylin and eosin (H&E). Histological changes were observed under light microscopy to assess the severity of lung inflammation and injury according to previously described criteria [20].

### Immunofluorescence staining

Lung tissues on day 7 p.i. were fixed with 4% PFA for 24 hours at 4°C, followed by gradient dehydration in 15% and 30% sucrose solutions for another 24 hours. The tissues were then embedded in Tissue-Tek O.C.T. Compound (SAKURA, Baltimore, MD, USA) and cryosectioned at a thickness of 8 μm. Sections were blocked with 10% goat serum at room temperature for 1 hour, then incubated with anti-Ly6G antibody (1:200 dilution, ab25377, abcam, Cambridge, England) overnight at 4°C, followed by incubation with Goat Anti-Rat IgG H&L (Alexa Fluor 488) preadsorbed secondary antibody (1:300 dilution, ab150165, abcam) for 1 hour at room temperature in the dark. After washing the sections were incubated with AutoFluoQuencher (APPLYGEN, Beijing, China) for 15 minutes and sealed with DAPI Fluoromount-G (SouthernBiotech, UAB, Birmingham, AL, USA). Images were acquired using a fluorescent microscope (200×).

### Lung and spleen single cells preparation

Infected lung samples were aseptically harvested at various time points and digested in RPMI 1640 (Gibco, GrandIsland, USA) containing 2 mg/mL collagenase XI (Sigma-Aldrich, St. Louis, MO, USA) for 55 minutes at 37°C, 5% CO_2_. During the final 5 minutes of digestion, 2 mM EDTA was added. After enzymatic digestion, 35% Percoll (GE Healthcare, Chicago, London, England) was added to the samples which were then centrifuged at 12°C, 2000 rpm for 20 min to remove tissue fascia. Spleen samples collected at the same time points were directly homogenized and filtered through a 70-μm cell strainers. Erythrocytes were lysed using ACK lysis buffer (Tris-NH_4_Cl), and the single-cell suspensions were resuspended in complete RPMI-1640 medium (RPMI-1640 supplemented with 10% heat inactivated fetal bovine serum (FBS, Shanghai Life iLab Biotech, Shanghai, China), 0.05 mmol/L 2-mercaptoethanol, 100 U/mL penicillin, and 0.1 mg/mL Streptomycin (Solarbio, Beijing, China). Cell concentrations were determined by trypan blue staining and adjusted to 2×10^6^ cells/mL for lung and 1×10^7^ cells/mL for spleen prior to subsequent experiments.

### Flow cytometry analysis

For surface staining, lung single cells were incubated with the Zombie NIR Fixable Viability Kit (423106, Biolegend) at room temperature in the dark for 15 minutes to exclued dead cells. After washing with PBS containing 2% FBS, the cells were then incubated with CD16/CD32 (Invitrogen, Carlsbad, CA, USA) for 30 minutes at 4°C in the dark to block non-specific Fc staining. The samples were divided and stained using anti-CD45-PerCp-Cy5.5, anti-F4/80-APC, anti-CD11c-FITC, anti-MHC II-PE, anti-Ly6C-PE-Cy7, anti-Ly6G-APC, anti-CD11b-FITC, anti-CD3-FITC, anti-CD4-APC, anti-CD8-PerCp-Cy5.5, anti-CD69-PE, anti-CD62L-PE-Cy7, and anti-CCR2-PE (all purchased from Biolegend, San Diego, CA, USA) for 30 minutes at 4°C in the dark to stain the macrophages, dendritic cells, monocytes, neutrophils, CD3^+^ T cells, CD4^+^ T cells, and CD8^+^ T cells as well as express CCR2 on cells.

For intracellular cytokine staining, lung and spleen single cells were stimulated with PMA (50 ng/mL, Sigma-Aldrich), ionomycine (1μg/mL, MCE, Monmouth Junction, NJ, USA), and brefeldin A (10 μg/mL, BioLegend, San Diego, CA, USA) for 5–6 hours at 37°C. Cells were first incubated with Zombie NIR Fixable Viability Kit to exclued dead cells. Fc segments were blocked with anti-CD16/32 and stained for surface antigens (anti-CD3-FITC, anti-CD4-APC, and anti-CD8-PerCp-Cy5.5). Then the cells were fixed in Fixation Buffer (Biolegend) in the dark at room temperature for 20 minutes followed by permeabilization using 1× Intracellular Staining Perm Wash Buffer (Biolegend) for 30 minutes. Finally, the cells were incubated with anti-IFN-γ-PE, anti-IL-4-PE-Cy7, and anti-IL-17A-PE antibodies for 40 min at room temperature in the dark.

For intranuclear Ki-67 and Foxp3 staining, cells were stained for surface antigens (anti-CD3-FITC, anti-CD4-APC, and anti-CD8-PerCp-Cy5.5), fixed, and permeabilized with 1× Fix/Perm Buffer (BD Biosciences) at 4°C for 50 min. After washing with 1× Perm/Wash Buffer, the cells were stained with anti-Ki-67-PE-Cy7 and anti-Foxp3-PE for 50 min at room temperature. Compensation for spectral mixing was applied by flow cytometric analysis of singly stained cells using antibodies against CD4 or CD3 labeled with APC-Cy7, PerCp-Cy5.5, APC, FITC, PE, and PE-Cy7. Flow cytometry analysis was conducted using a FACS Canto II flow cytometer (BD Biosciences, Franklin, NJ, USA), and the raw flow cytometry data were analyzed using FlowJo version 10.

### Enzyme-linked immunosorbent assay (ELISA)

Lung single cells were cultured at a concentration of 7.5×10^6^ cells/well in 96-well plates, with or without stimulation by UV-inactivated *C*. *muridarum* (1×10^5^ IFUs/mL) at 37°C in 5% CO_2_ incubator for 48 hours. After incubation, culture supernatants were collected, and the levels of IFN-γ, IL-4, and IL-17A were measured by ELISA. For each cytokine, purified capture antibodies (IFN-γ, 14-7313-85, eBioscience; IL-4, 554434, BD; IL-17A, 14-7175-85, eBioscience) were coated onto plates overnight. After blocking with 1% BSA for 1 to 2 hours at room temperature, supernatants were added and incubated at 37°C for 2.5 to 3 hours. Detection was performed using specific biotinylated antibodies (IFN-γ, 13-7312-81, eBioscience; IL-4, 554390, BD; IL-17A, 13-7177-85, eBioscience), followed by incubation Alkaline Phosphatase-conjugated Streptavidin (Jackson Immuno Research, 016-050-084) at 37°C for 45 minutes. Phosphatase substrate (S0942-200TAB, Sigma) was then added and incubated for 30–40 minutes at room temperature. Absorbance was measured at 405 nm using a microplate reader, and the concentrations of IFN-γ, IL-4, and IL-17A were calculated based on standard curves.

### Protein extraction and Western blot analysis

Total protein was extracted from lung tissues on day 7 p.i. using RIPA lysis buffer (R0278, Millipore, Massachusetts, USA) containing 1× protease and phosphatase inhibitor cocktail (78442, Thermo Fisher Scientific, USA). Protein concentrations were determined via the bicinchoninic acid (BCA) assay (Thermo Fisher Scientific). Equal amounts of protein lysates were separated by 10% sodium dodecyl sulfate-polyacrylamide gel electrophoresis (SDS-PAGE) and transferred to polyvinylidene fluoride (PVDF) membranes. The membranes were blocked with TBST buffer containing 5% BSA for 2 hours at room temperature, then incubated overnight at 4°C with primary antibodies: Stat1 (D1K9Y) Rabbit mAb (14994S), Phospho-Stat1 (Tyr701) (58D6) Rabbit mAb (9167S), Stat6 Antibody (9362S), Phospho-Stat6 (Tyr641) (D8S9Y) Rabbit mAb (56554S) (Cell Signaling Technology, Massachusetts, USA) and β-actin Rabbit mAb (High Dilution) (A2235, ABclonal, Wuhan, China). After washing, the blots were incubated with Goat anti-Rabbit IgG-HRP Antibody (abs20040ss, Absin, Shanghai, China) for 1 hour at room temperature. Protein bands were visualized using the StarSignal High sensitivity chemiluminescence kit (GenStar, Beijing, China) and imaged with the Tanon-5200 Imager (Tanon, Shanghai, China). Densitometry was performed using ImageJ software for quantification.

### Statistical analysis

GraphPad Prism 10 (GraphPad InStat Software, San Diego, CA, USA) was used for statistical analysis. Differences between two different groups were assessed using two-way ANOVA. Differences among multiple groups were analyzed by one-way ANOVA. All data results were presented as mean ± SD. *p* < 0.05 was considered statistically significant (* *p* < 0.05; ** *p* < 0.01; *** *p* < 0.001; **** *p* < 0.0001).

## Results

### Elevated expression of CCR2 during *C*. *muridarum* respiratory infection

**Fig 1:**
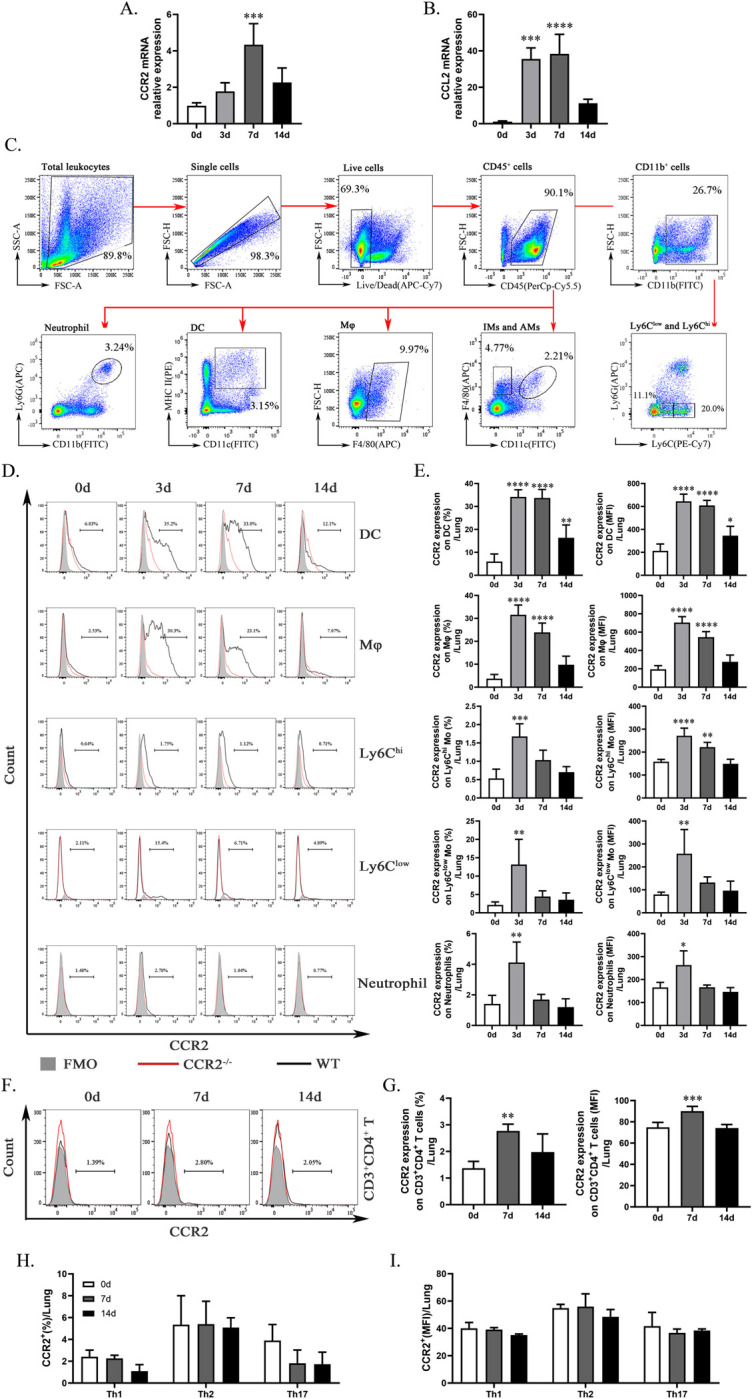
*C*. *muridarum* respiratory infection induces CCR2 expression on lung tissue and immune cells. The C57BL/6 mice were infected with 1 × 10^3^ inclusion forming units (IFUs) of Chlamydia muridarum (*C*. *muridarum*) via the respiratory tract. Lung tissue was retrieved from infected animals on days 0, 3, 7, and 14 post infection (p.i.). (A and B) Total RNA extracted from the lung tissue was assayed for *ccr2* (A) and *ccl2* (B) mRNA expression by quantitative real-time PCR (qPCR). (C) The flow cytometry gating strategy for lung immune cells. Live cells from the lungs of mice are first selected based on their morphology using FSC/SSC parameters, followed by the exclusion of doublets and dead cells. Neutrophils are presented as CD45^+^CD11b^+^Ly6G^+^ cells. Dendritic cells (DC) are presented as CD45^+^MHCII^+^CD11c^+^cells. Macrophages (Mφ) are presented as CD45^+^F4/80^+^ cells. Alveolar macrophages (AMs) are presented as CD45^+^F4/80^+^CD11c^hi/+^ cells. Interstitial macrophages (IMs) are presented as CD45^+^F4/80^+^CD11c^low/-^ cells. Ly6C^hi^ monocytes (Ly6C^hi^ Mo) are presented as CD45^+^CD11b^+^Ly6G^-^Ly6C^hi^ cells, Ly6C^low^ monocytes (Ly6C^low^ Mo) are presented as CD45^+^CD11b^+^Ly6G^-^Ly6C^low^ cells. (D and E) The figures show representative histograms of CCR2 intensity. Black line: wild type mice; red line: naïve *ccr2*-deficient mice; grey histogram: fluorescence minus one (FMO). Representative flow cytometric images (D), along with the percentages (E, left) and mean fluorescence intensity (MFI) (E, right) of CCR2 on DC, Mφ, Ly6C^hi^ Mo, Ly6C^low^ Mo, and neutrophils, are shown. (F and G) The figures show representative histograms of CCR2 intensity. Black line: wild type mice; red line: naïve *ccr2*-deficient mice; grey histogram: FMO. Representative flow cytometric images (F), along with the percentages (G, left) and MFI (G, right) of CCR2 on CD3^+^CD4^+^ T cells, are shown. (H and I) The percentages (H) and MFI (I) of CCR2 on Th1 cells, Th2 cells, and Th17 cells are depicted. Data are presented as means ± SD from n = 3–5 per time point, representative of one of three independent experiments. Statistical significance of differences was determined by one-way ANOVA. * *p* < 0.05, ** *p* < 0.01, *** *p* < 0.001, **** *p* < 0.0001.

Research on CCR2 has predominantly focused on monocyte migration, with limited understanding of its role in intracellular bacterial infections. To address this knowledge gap, we established a Chlamydia respiratory infection model in mice to investigate the impact of CCR2 on these infections. As shown in Fig 1A and 1B, *C*. *muridarum* infection induces an upregulation of *ccr2* expression in the lungs, which peaks on day 7 p.i.. Similarly, *ccl2* mRNA levels significantly increased on day 3 p.i., peaked on day 7 p.i., and then declined by day 14 p.i.. We further analyzed the surface expression of CCR2 on various pulmonary immune cells following *C*. *muridarum* infection. Fig 1C illustrates the flow cytometry gating strategy for dendritic cell (DC, CD45^+^CD11c^+^MHCII^+^), macrophage (Mφ, CD45^+^F4/80^+^), alveolar macrophages (AMs, CD45^+^F4/80^+^CD11c^hi/+^), interstitial macrophages (IMs, CD45^+^F4/80^+^CD11c^low/-^), classical monocytes (Ly6C^hi^ Mo, CD11b^+^Ly6G^-^Ly6C^hi^), non-classical monocytes (Ly6C^low^ Mo, CD11b^+^Ly6G^-^Ly6C^low^), and neutrophils (CD45^+^CD11b^+^Ly6G^+^). Flow cytometry results revealed basal CCR2 expression on DC, Mφ, Ly6C^hi^ Mo, Ly6C^low^ Mo, and neutrophils with a notable induction of CCR2 expression upon infection (Fig 1D and 1E). On days 3 and 7 p.i., CCR2 expression remained consistently elevated on DC and Mφ, whereas Ly6C^low^ Mo, Ly6C^hi^ Mo, and neutrophils exhibited higher expression only on day 3 p.i.. Notably, CCR2^+^Ly6C^hi^ Mo and CCR2^+^ neutrophils comprised a relatively small proportion of total lung cells, suggesting that CCR2 may play a regulatory role in specific myeloid populations, particularly DC, Mφ, and Ly6C^low^ Mo. We subsequently investigated the expression of CCR2 on CD4^+^ T cells following *C*. *muridarum* respiratory infection. As shown in Fig 1F and 1G, basal CCR2 expression on CD3^+^CD4^+^ T cells was low, consistent with reports that naïve CD4^+^ T cells in peripheral tissues (spleen, lymph nodes, and lungs) express minimal CCR2 [22,23]. On day 7 p.i., both the percentage of CCR2^+^CD3^+^CD4^+^ T cells and the mean fluorescence intensity (MFI) of CCR2 on CD3^+^CD4^+^ T cells increased significantly, returning to baseline by day 14 p.i. (Fig 1F and 1G). Further analysis showed that CCR2 expression on Th1, Th2, and Th17 cells remained low and was not significantly altered by *C*. *muridarum* infection (Fig 1H and 1I).

These findings suggest that CCR2 orchestrates complex and distinct regulatory mechanisms in both innate and adaptive immune responses during *C*. *muridarum* respiratory infection. Specifically, CCR2 selectively modulates distinct myeloid populations, and its regulatory effect on Th1/Th2/Th17 responses is likely indirect.

### *Ccr2*^-/-^ mice exhibited reduced resistance and severe inflammation in response to *C*. *muridarum* respiratory infection

**Fig 2:**
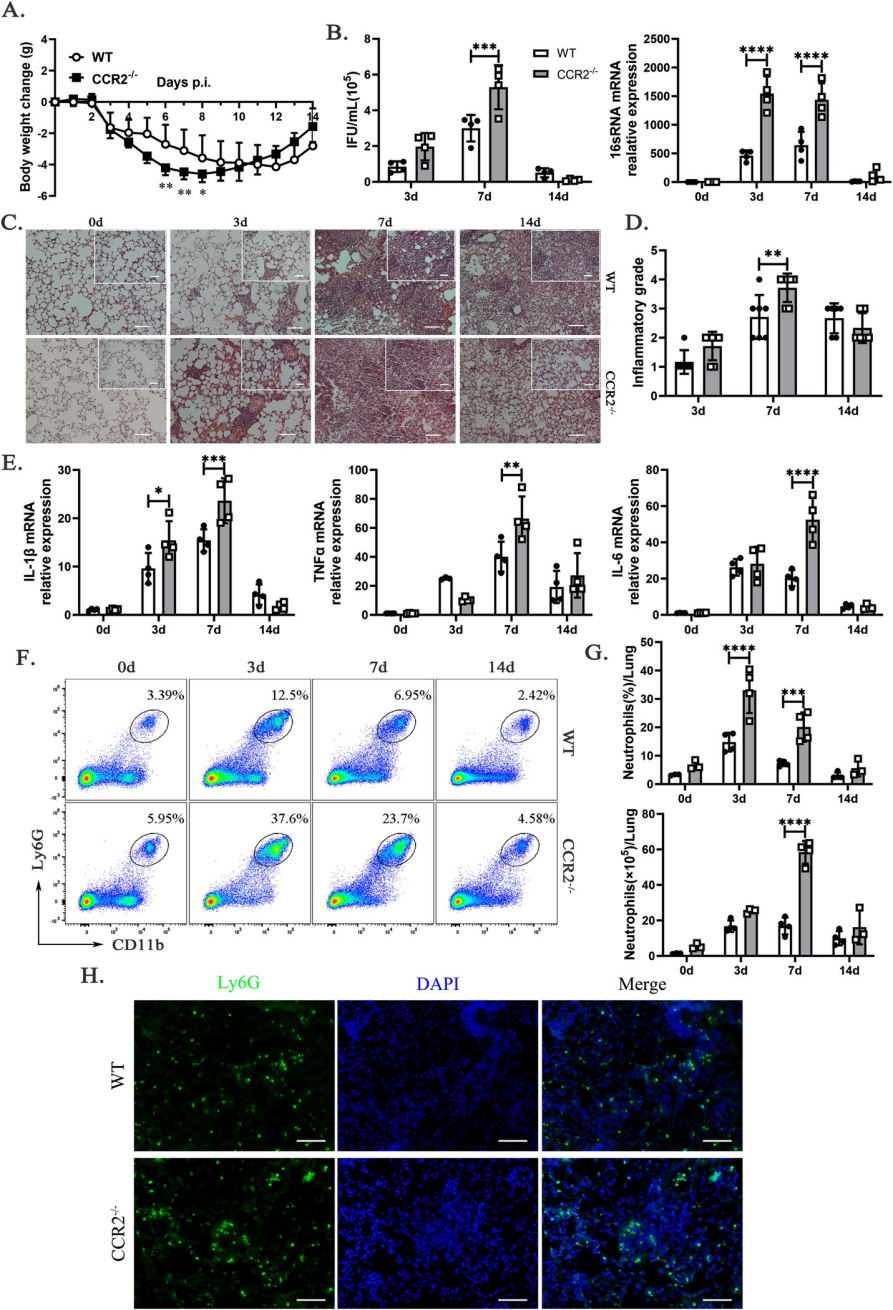
Reduced resistance and exacerbated lung inflammation in *ccr2*^-/-^ mice with *C*. *muridarum* respiratory infection. WT and *ccr2*-deficient (*ccr2*^-/-^) mice were infected with 1×10^3^ IFUs of *C*. *muridarum* via the respiratory tract and sacrificed on days 0, 3, 7, and 14 p.i.. (A) Weight changes in WT and *ccr2*^-/-^ mice following *C*. *muridarum* respiratory tract infection were monitored for 14 days. (B) Chlamydial burdens in the lungs were determined by quantifying IFUs (left) and *16S rRNA* mRNA expression (right). (C) Representative images of H&E-stained lung sections from each group were observed under light microscopy at magnifications of ×100 (scale bar, 200 μm) and ×200 (top right; scale bar, 100 μm). (D) Inflammatory grades were assessed based on the H&E staining results, as described in the Materials and Methods. (E) Relative mRNA expressions levels of *il-1β*, *tnfα*, and *il-6* were quantified by qPCR in lung tissues from different groups, normalized to *β-actin*. (F) Representative flow cytometry images of neutrophils (Ly6G^+^CD11b^+^ cells) from lung tissues of WT and *ccr2*^-/-^ mice infected with *C*. *muridarum* on different days. (G) Flow cytometry data showing the percentages (above) and absolute numbers (below) of neutrophils in lung tissues of WT and *ccr2*^-/-^ mice infected with *C*. *muridarum* on different days. (H) Representative immunofluorescence images of Ly6G^+^ cells in lung tissues from WT and *ccr2*^-/-^ mice on day 7 p.i. (Green, Ly6G^+^ cells; Blue, DAPI) (×200; scale bar, 100 μm). Data are presented as means ± SD from n = 3–7 mice per genotype and time point, representative of one of three independent experiments. Statistical significance of differences was determined by two-way ANOVA. * *p* < 0.05, ** *p* < 0.01, *** *p* < 0.001, **** *p* < 0.0001.

To clarify the relationship between CCR2 and the clearance of chlamydial infections, we established a respiratory infection model using WT and *ccr2*-deficient (*ccr2*^-/-^) mice. The results indicate that *C*. *muridarum*-infected *ccr2*^-/-^ mice developed a more severe and rapidly progressing disease compared to WT mice. Both mouse strains began losing weight from day 3 p.i., but *ccr2*^-/-^ mice experienced greater weight loss from days 3 to 10 p.i., with a significantly sharper decline from days 6 to 8 p.i. and a faster recovery from days 11 to 14 p.i. (Fig 2A). Evaluation of Chlamydia loads in the lungs, measured by IFUs and *16S rRNA* expression, revealed significantly impaired clearance in *ccr2*^-/-^ mice, with higher levels compared to WT mice on day 7 p.i. (Fig 2B). Histopathological examination of the lungs from *ccr2*^-/-^ mice revealed significant tissue damage, pathological changes, and infiltration of inflammatory cells (Fig 2C). A higher inflammatory grade was observed on day 7 p.i. in *C*. *muridarum*-infected mice compared to WT mice, which gradually decreased by day 14 p.i. (Fig 2D). Additionally, infected *ccr2*^-/-^ mice displayed elevated levels of pro-inflammatory cytokines, including *il-1β*, *tnfα*, and *il-6*, on days 7 p.i. compared to WT mice (Fig 2E). Notably, *il-1β* mRNA levels in the lungs of *ccr2*^-/-^ mice were significantly increased as early as day 3 p.i. (Fig 2E). Neutrophils, which are known to drive inflammatory responses, were significantly more abundant in *ccr2*^-/-^ mice than in WT mice on days 3 and 7 p.i., with markedly higher absolute cell numbers in *ccr2*^-/-^ mice on day 7 p.i. (Fig 2F and 2G). This increase was confirmed by Ly6G-positive immunofluorescence staining, which demonstrated a marked elevation of neutrophils in the lungs of *ccr2*^-/-^ mice on day 7 p.i. (Fig 2H).

These results indicate that CCR2 deficiency significantly exacerbates susceptibility to *C*. *muridarum* respiratory infections by impairing pathogen clearance and intensifying inflammation. The CCR2-mediated immune response is important for effective host resistance, particularly during the mid-phase of infection (day 7 p.i.), highlighting its critical role in managing both the inflammatory response and pathogen clearance.

### CCR2 deficiency hinders myeloid and T cell infiltration and impairs T cell activity during *C*. *muridarum* respiratory infection

**Fig 3:**
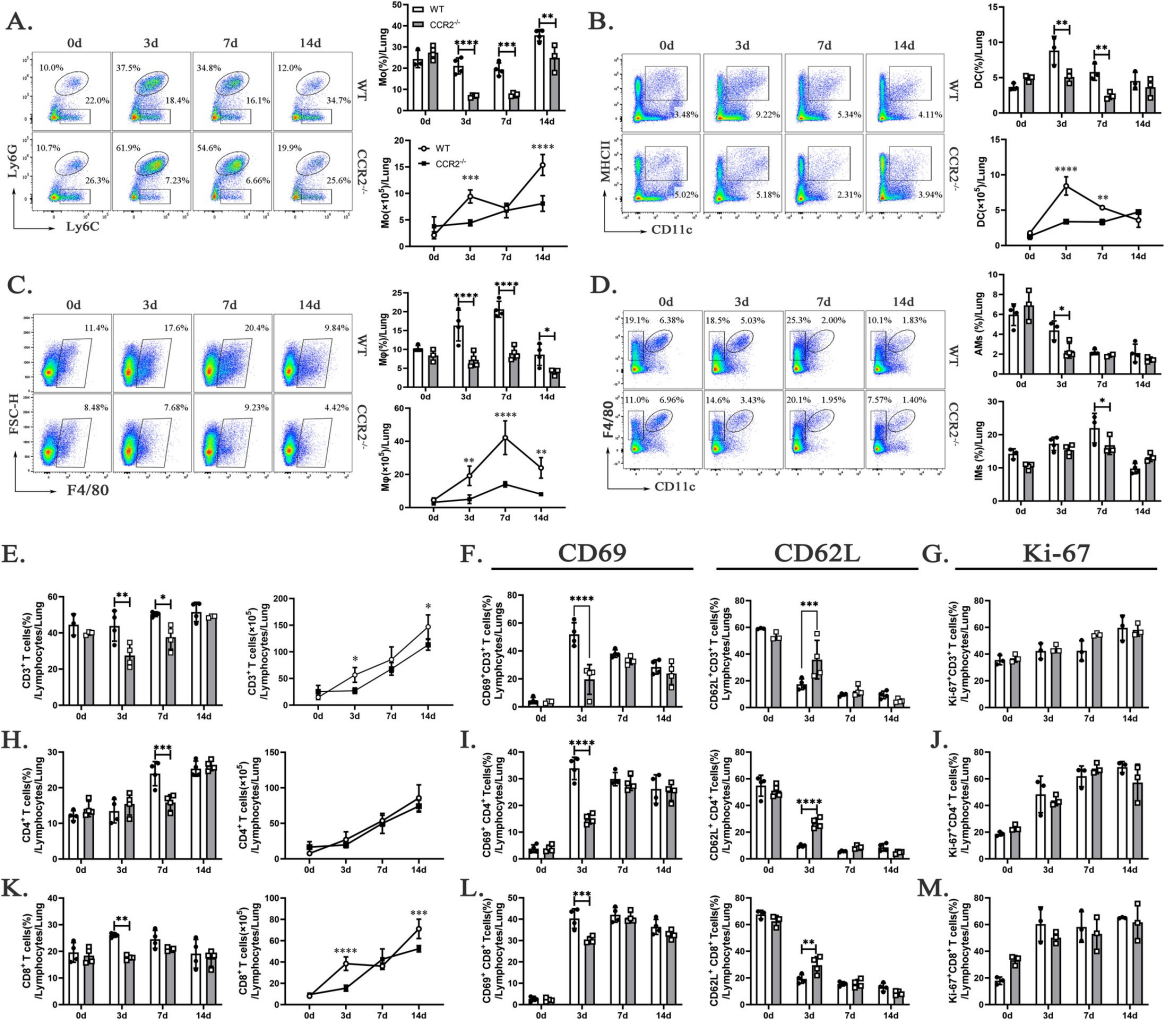
CCR2 deficiency attenuates the infiltration of myeloid cells and diminishes the numbers and activation of T cells following *C*. *muridarum* respiratory infection. (A—D) Representative flow cytometry images (left), percentages (upper right), and absolute numbers (lower right) of Mo (A), DC (B), Mφ (C), AMs, and IMs (D) from lung tissues of WT and *ccr2*^-/-^ mice infected with *C*. *muridarum* on different days. (E, H, and K) Flow cytometry data were summarized to show the percentages (left) and absolute numbers (right) of CD3^+^ T cells (E), CD4^+^ T cells (H), and CD8^+^ T cells (K) from lung tissues of WT and *ccr2*^-/-^ mice infected with *C*. *muridarum* on different days. (F, I, and L) Flow cytometry data were summarized to show the percentages of CD69^+^CD3^+^ T cells, CD62L^+^CD3^+^ T cells (F), CD69^+^CD4^+^ T cells, CD62L^+^CD4^+^T cells (I), CD69^+^CD8^+^ T cells, and CD62L^+^CD8^+^T cells (L) from lung tissues of WT and *ccr2*^-/-^ mice infected with *C*. *muridarum* on different days. (G, J, and M) Flow cytometry data were summarized to show the percentages of Ki-67^+^CD3^+^ T cells (G), Ki-67^+^CD4^+^ T cells (J), and Ki-67^+^CD8^+^ T cells (M) from lung tissues of WT and *ccr2*^-/-^ mice infected with *C*. *muridarum* on different days. Data are presented as means ± SD from n = 3–4 mice per genotype and time point, representative of one of three independent experiments. Statistical significance of differences was determined by two-way ANOVA. * *p* < 0.05, ** *p* < 0.01, *** *p* < 0.001, **** *p* < 0.0001.

To elucidate the mechanisms underlying infection susceptibility in *ccr2*^-/-^ mice, we assessed the temporal accumulation of myeloid cells in the lung following *C*. *muridarum* respiratory infection. Flow cytometry results demonstrated a significant reduction in myeloid cell populations in the lungs of *ccr2*^-/-^ mice, including Mo, DC, Mφ, AMs, and IMs (Fig 3A–3D). This reduction of myeloid innate immune cells likely contributed to impaired adaptive immune responses, as demonstrated by a persistent and significant decrease in the accumulation of CD3^+^ T cells in the lungs of *ccr2*^-/-^ mice (Fig 3E). The reduced T cell numbers were accompanied by diminished activity, indicated by a decrease in CD69^+^CD3^+^ T cells and an increase in CD62L^+^CD3^+^ T cells on day 3 p.i. (Fig 3F), although their proliferative capacity, measured by Ki-67 expression, remained unaffected compared to WT mice (Fig 3G). Moreover, a significant decrease in the percentage of CD4^+^ T cells was observed in *ccr2*^-/-^ mice on day 7 p.i., although their absolute numbers remained unchanged (Fig 3H). In contrast, the percentage of CD8^+^ T cells in *ccr2*^-/-^ mice exhibited a significant decline as early as day 3 p.i., accompanied by a marked reduction in their absolute numbers on days 3 and 14 p.i. (Fig 3K). Importantly, despite no significant alterations in their proliferative capacity (Fig 3J and 3M), the activity of CD4^+^ T cells and CD8^+^ T cells was markedly reduced on day 3 p.i. (Fig 3I and 3L). These results underscore the pivotal role of chemokine signaling in orchestrating immune cell recruitment and activation. The increased susceptibility of *ccr2*^-/-^ mice to *C*. *muridarum* respiratory infection is linked to a profound impairment in the accumulation and functional activity of both innate and adaptive immune cells. This compromised immune cell dynamics not only exacerbates infection severity but also suggests a disrupted balance in the host’s immune response, setting the stage for a deeper investigation into the underlying T cell-mediated immune mechanisms.

### CCR2 deficiency impairs the differentiation of Th1 cells and the secretion of IFN-γ in *C*. *muridarum*-infected lung

**Fig 4:**
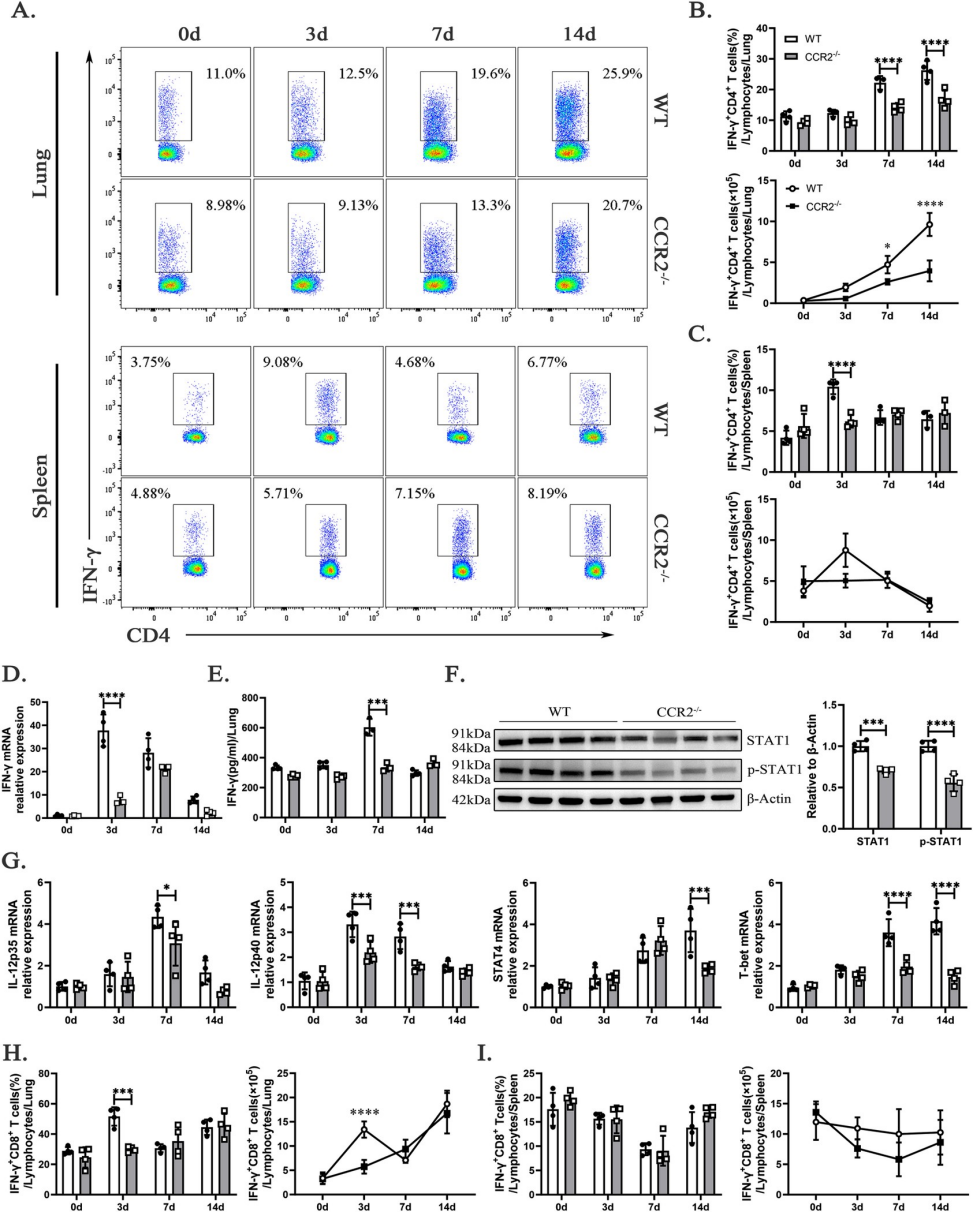
Th1 differentiation and IFN-γ secretion are impaired in *ccr2*^-/-^ mice during *C*. *muridarum* infection. (A) Live cells from the lungs and spleens of mice were first selected based on their morphology using FSC/SSC parameters, followed by the exclusion of doublets and dead cells. Representative intracellular cytokine staining images of Th1 cells (CD3^+^CD4^+^IFN-γ^+^ T cells) from the lungs and spleens of WT and *ccr2*^-/-^ mice infected with *C*. *muridarum* on different days are shown. (B and C) Flow cytometry data were summarized to show the percentages (above) and absolute numbers (below) of Th1 cells in the lungs (B) and spleens (C) of WT and *ccr2*^-/-^ mice infected with *C*. *muridarum* on different days. (D) Relative mRNA expression levels of IFN-γ were quantified by qPCR in lung tissues from the different groups after normalization to *β-actin*. (E) Levels of INFγ in the supernatants of cultured lung cells were measured by enzyme-linked immunosorbent assay (ELISA) in the different groups. (F) The protein expression levels of STAT1 and p-STAT1 were quantified by Western blotting in the lung tissues of WT and *ccr2*^-/-^ mice on day 7 p.i.. On the left is a representative Western blotting image, and on the right is a statistical graph. (G) Relative mRNA expressions of *il-12p35*, *il-12p40*, *stat4*, and *T-bet* were quantified by qPCR in lung tissues from the different groups after normalization to *β-actin*. (H and I) Flow cytometry data were summarized to show the percentages (left) and absolute numbers (right) of IFN-γ^+^CD8^+^ T cells in the lungs (H) and spleens (I) of WT and *ccr2*^-/-^ mice infected with *C*. *muridarum* on different days. Data are presented as means ± SD from n = 3–4 mice per genotype and time point, representative of one of three independent experiments. Statistical significance of differences was determined by two-way ANOVA. * *p* < 0.05, *** *p* < 0.001, **** *p* < 0.0001.

Th1 cells, a subset of CD4^+^ T cells that secrete type 1 cytokines, are essential for clearing Chlamydial infections by producing IFN-γ and enhancing the protective functions of immune and inflammatory cells [1,24]. Chemokines and their receptors play a crucial role in regulating leukocyte recruitment and shaping T-cell responses, prompting us to investigate their involvement in modulating Th1 cell development and influencing infection outcomes. As shown in Fig 4A–4C, the percentages and absolute numbers of CD3^+^CD4^+^IFN-γ^+^ T cells (Th1 cells) in the lungs of *ccr2*^-/-^ mice were significantly lower than those in WT mice on days 7 and 14 p.i., and the percentages of Th1 cells in the spleen were also significantly reduced on day 3 p.i.. The expression of *ifn-γ* mRNA in lung tissue from *ccr2*^-/-^ mice was significantly decreased on day 3 p.i. (Fig 4D), followed by a reduction in IFN-γ secretion by lung cells on day 7 p.i. (Fig 4E). This decrease in IFN-γ production by T cells, which are critically dependent on STAT1 for functional activity, was accompanied by downregulated STAT1 expression and reduced STAT1 phosphorylation in infected lung tissue from *ccr2*^-/-^ mice on day 7 p.i. (Fig 4F). Furthermore, we observed attenuated expression of Th1-promoting cytokines *il-12p35* and *il-12p40*, along with decreased levels of the Th1-associated transcription factors *stat4* and *T-bet*, in lung tissue from *C*. *muridarum-*infected *ccr2*^-/-^ mice (Fig 4G).

Cytotoxic T lymphocytes (CTLs), a key source of IFN-γ, play a crucial role in bacterial clearance and contribute to protective immune responses against respiratory tract chlamydial infections [25,26]. In this study, we observed a notable reduction in CD8^+^ T cells in the lungs of *ccr2*^-/-^ mice following infection (Fig 3K). In particular, we found a significant decrease in both the percentage and absolute number of IFN-γ^+^CD8^+^ T cells in the lungs of *ccr2*^-/-^ mice compared to WT mice on day 3 p.i. (Fig 4H). In contrast, no significant differences were detected in the percentage or absolute number of IFN-γ^+^CD8^+^ T cells in the spleen (Fig 4I). These findings highlight the critical role of CCR2 in recruiting or maintaining CTLs in the pulmonary environment during *C*. *muridarum* infection.

In conclusion, these findings emphasize the impaired Th1 cell differentiation and IFN-γ secretion in *ccr2*^-/-^ mice, further underscoring the critical role of CCR2 in modulating the host response to pulmonary *C*. *muridarum* infection. The absence of CCR2 exacerbates *C*. *muridarum* infection in the respiratory tract by impairing the differentiation of CD4^+^ T cells into Th1 cells and reducing IFN-γ secretion, thereby weakening the immune response to Chlamydia infection.

### Excessive Th2-type immune response in CCR2 deficiency promotes lung pathology and increases *C*. *muridarum* respiratory infection susceptibility

**Fig 5:**
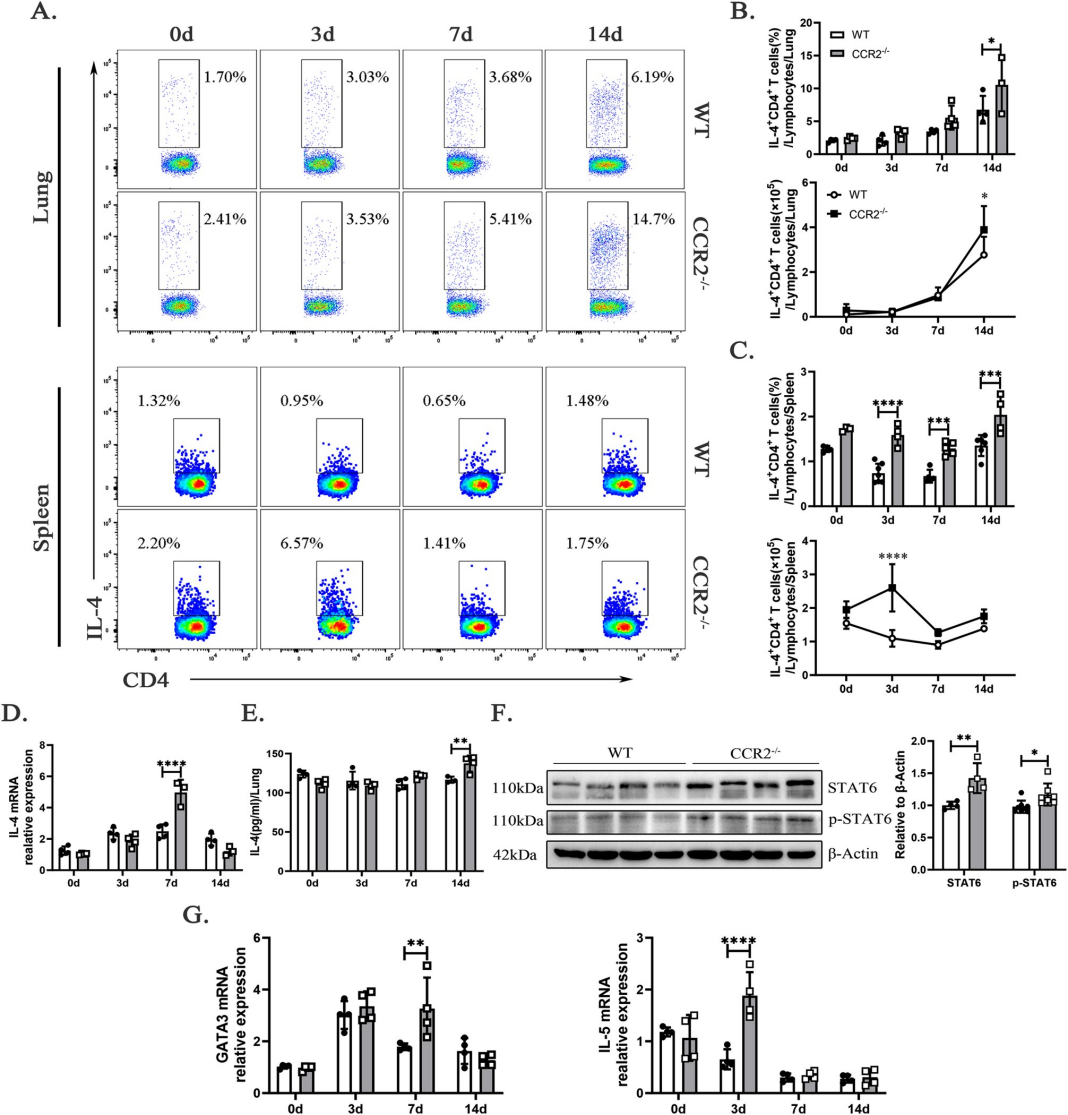
CCR2 deficiency upregulates IL-4, IL-5, and Th2 transcription factors during *C*. *muridarum* infection. (A) Representative intracellular cytokine staining images of Th2 cells (CD3^+^CD4^+^IL-4^+^ T cells) from the lungs and spleens of WT and *ccr2*^-/-^ mice infected with *C*. *muridarum* on different days. (B and C) Flow cytometry data were summarized to show the percentages (above) and absolute numbers (below) of Th2 cells in the lungs (B) and spleens (C) of WT and *ccr2*^-/-^ mice infected with *C*. *muridarum* on different days. (D and G) Relative mRNA expression levels of *il-4* (D), *gata3*, and *il-5* (G) were quantified by qPCR in lung tissues from the different groups after normalization to *β-actin*. (E) Levels of IL-4 in lung cell culture supernatants were measured by ELISA from the different groups. (F) The protein expression levels of STAT6 and p-STAT6 were quantified by Western blotting in the lung tissue of WT and *ccr2*^-/-^ mice on day 7 p.i.. On the left is a representative image, and on the right is a statistical graph. Data are presented as means ± SD from n = 3–7 mice per genotype and time point, representative of one of three independent experiments. Statistical significance of differences was determined by two-way ANOVA. * *p* < 0.05, ** *p* < 0.01, *** *p* < 0.001, **** *p* < 0.0001.

An excessive Th2-type response can impair host resistance to intracellular Chlamydia infection [1,27], prompting us to examine IL-4, a key driver of Th2 differentiation [28], and evaluate its role in *ccr2*^-/-^ mice. As shown in Fig 5A–5C, the percentages and absolute numbers of CD3^+^CD4^+^IL-4^+^ T cells (Th2 cells) in the lungs of *ccr2*^-/-^ mice were significantly higher than those in WT mice on day 14 p.i., and the percentages of Th2 cells in the spleen were also significantly increased on days 3, 7, and 14 p.i. The elevated expression of *il-4* mRNA in the lung tissue of *ccr2*^-/-^ mice on day 7 p.i. (Fig 5D) and increased IL-4 secretion (Fig 5E) from lung cells on day 14 p.i. support the idea that CCR2 deficiency skews the immune response toward Th2 polarization. This is further confirmed by the activation of the STAT6-GATA3 signaling pathway, which is essential for Th2 differentiation, as indicated by heightened levels of STAT6 expression and phosphorylation in the infected lung tissue of *ccr2*^-/-^ mice on day 7 p.i. (Fig 5F). Importantly, the elevated levels of *gata3* and heightened production of *il-5* (Fig 5G) indicate an upregulated Th2 response in *ccr2*^-/-^ mice, potentially compromising their capacity to mount an efficient immune defense against *C*. *muridarum*. These data indicate that in *ccr2*^-/-^ mice, *C*. *muridarum* infection elicits a significant Th2-skewed immune response, characterized by elevated production of *il-4* and *il-5*, along with enhanced expression of Th2-promoting transcription factors, potentially compromising the host’s ability to combat the infection effectively.

### Excessive Th17-derived IL-17 drives neutrophil-mediated lung inflammation in *ccr2*^*-/-*^ mice during *C*. *muridarum* infection

**Fig 6:**
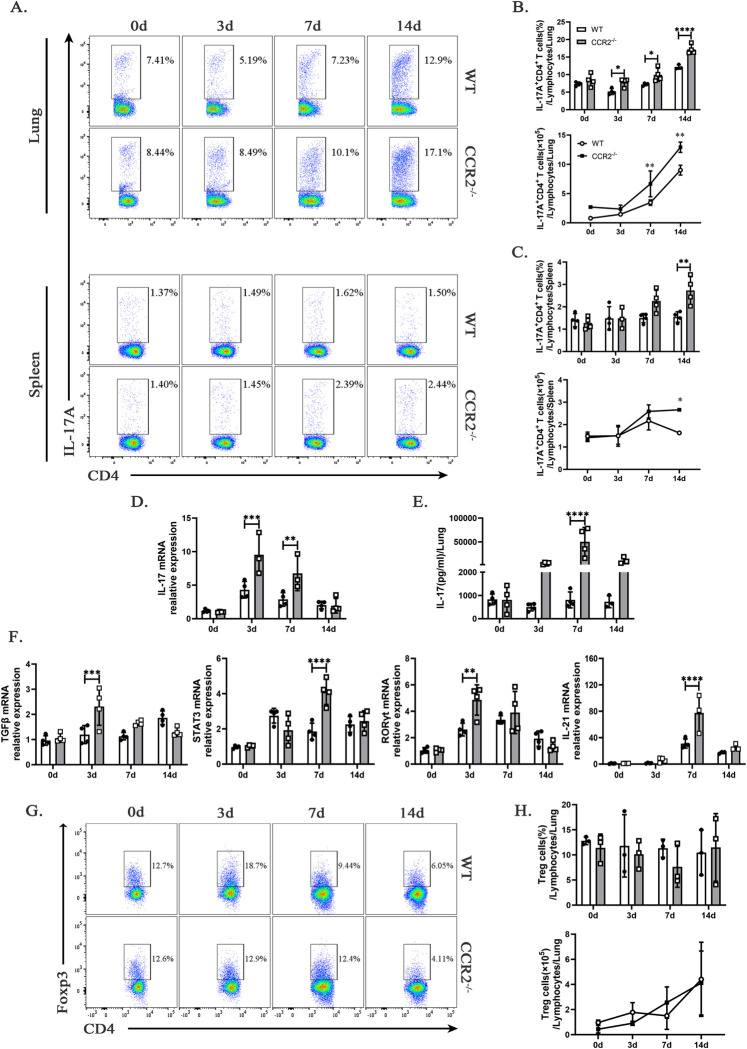
CCR2 deficiency elevates *C*. *muridarum*-induced Th17-type immune response. (A) Representative flow cytometry images of Th17 cells (CD3^+^CD4^+^IL-17A^+^ T cells) from the lungs and spleens of WT and *ccr2*^-/-^ mice infected with *C*. *muridarum* on different days. (B and C) Flow cytometry data were summarized to show the percentages (above) and absolute numbers (below) of Th17 cells in the lungs (B) and spleens (C) of WT and *ccr2*^-/-^ mice infected with *C*. *muridarum* on different days. (D and F) Relative mRNA expressions of *il-17* (D), *TGFB*, *stat3*, *RORγt*, and *il-21* (F) were quantified by qPCR in lung tissues from the different groups after normalization to *β-actin*. (E) Levels of IL-17 in lung cell culture supernatants were measured by ELISA from the different groups. (G) Representative flow cytometry images of Treg cells (CD3^+^CD4^+^Foxp3^+^ T cells) from the lungs of WT and *ccr2*^-/-^ mice infected with *C*. *muridarum* on different days. (H) Flow cytometry data were summarized to show the percentages (above) and absolute numbers (below) of Treg cells in the lungs of WT and *ccr2*^-/-^ mice infected with *C*. *muridarum* on different days. Data are presented as means ± SD from n = 3–4 mice per genotype and time point, representative of one of three independent experiments. Statistical significance of differences was determined by two-way ANOVA. * *p* < 0.05, ** *p* < 0.01, *** *p* < 0.001, **** *p* < 0.0001.

Our previous research has confirmed that IL-17/Th17 promotes type 1 T cell immunity against pulmonary intracellular bacterial infections by modulating DC function [25]. However, excessive IL-17 produced by Th17 cells mediates neutrophil recruitment in the lungs, leading to exacerbated lung inflammation during *C*. *muridarum* respiratory infection [20]. Fig 2F–2H show that *ccr2* deficiency leads to increased neutrophil infiltration in the lungs, suggesting a potential link between Th17-derived IL-17 production and neutrophil accumulation. As shown in Fig 6A–6C, *ccr2*^-/-^ mice exhibited significantly higher percentages of CD3^+^CD4^+^IL-17A^+^ T cells (Th17 cells) in the lungs on days 3, 7, and 14 p.i, while the absolute numbers of Th17 cells were elevated on days 7 and 14 p.i.. Similarly, an increase in both the percentage and absolute number of Th17 cells was observed in the spleen on day 14 p.i.. Elevated *il-17* mRNA expression and secretion in the lungs following *C*. *muridarum* infection support an enhanced Th17-type immune response in *ccr2*^-/-^ mice (Fig 6D and 6E). The upregulation of Th17-promoting *TGFB* mRNA on day 3 p.i. (Fig 6F) highlights its role in driving Th17 differentiation via STAT3 activation of RORγt, a key regulator of IL-17A production [29]. Quantification of *stat3* mRNA levels using qPCR revealed a significant increase in STAT3 expression in *C*. *muridarum*-infected *ccr2*^-/-^ mice compared to WT mice on day 7 p.i. (Fig 6F). This upregulation was accompanied by a rise in *RORγt* mRNA expression in the lungs of *ccr2*^-/-^ mice on day 3 p.i. (Fig 6F), underscoring the impact of CCR2 signaling on Th17 differentiation. Consistent with these findings, the Th17-derived cytokine *il-21* mRNA expression in the lungs of *ccr2*^-/-^ mice was also elevated on day 7 p.i.. Interestingly, there were no notable differences observed in the frequencies of Foxp3^+^ regulatory T cells between WT and *ccr2*^-/-^ mice (Fig 6G and 6H).

In conclusion, CCR2 deficiency amplifies the Th17-type immune response, resulting in increased IL-17 and IL-21 production and enhanced Th17 cell differentiation. This is associated with elevated neutrophil infiltration in the lungs during *C*. *muridarum* infection, highlighting the role of CCR2 in modulating Th17-driven inflammation and neutrophil recruitment, thereby exacerbating lung inflammation, while having no impact on Treg cells.

## Discussion

Chemokines bind to their specific receptors, triggering a cascade of intracellular signaling pathways that regulate the migration, localization, and activation of immune cells to sites of infection or inflammation. Research has confirmed that MIP-1α response is closely association with Th1 response in chlamydial lung infections [1]. In this study, we investigated the role of CCR2 in the host response to *C*. *muridarum* respiratory infection, revealing its critical involvement in regulating both innate and adaptive immune responses. The elevated expression of CCR2 in lung tissue and various immune cell populations, including DCs, Mφ, and monocytes, underscores its potential role in facilitating the recruitment and activation of immune cells during infection. Notably, CCR2 protein expression peaked in all analyzed cells on day 3 post-infection (p.i.), while a significant increase in mRNA levels in lung tissue was not observed until day 7 p.i.. We speculate that this phenomenon may be attributed to the measurement of CCR2 mRNA levels in whole lung tissue, which predominantly comprises epithelial cells, parenchymal cells, and fibroblasts—cell types that are generally not known to express CCR2. Although CCR2 is significantly induced in DC, Mφ, and Mo by day 3 p.i., the inflammation in the lung tissue remains mild, with limited immune cell infiltration at this stage, which likely explains the modest, statistically non-significant increase in CCR2 mRNA on day 3 p.i. compared to day 0. By day 7 p.i., significant immune cell infiltration into the lung is evident (Fig 2C and 2D), coinciding with a substantial upregulation of CCR2 mRNA expression.

Other studies have established that CCR2 expression in T cells is highest in the bone marrow (BM) compared to the spleen, lymph nodes, and lung [23]. Our results demonstrate that CCR2 expression on naïve CD4^+^ T cells is initially low and significantly upregulated by day 7 p.i. following *C*. *muridarum* respiratory infection (Fig 1F and 1G), although the expression remains at a relatively low level. These findings robustly highlight the role of CCR2 in the CD4^+^ T cell-mediated host anti-Chlamydia immune response. They also suggest that CCR2 is unlikely to serve as the primary or direct regulatory target in CD4^+^ T cell responses. Other studies have demonstrated that CCR2 is expressed on Th1, Th2, and Th17 cells, potentially influencing their differentiation [19]. To further explore this during Chlamydia respiratory infection, we analyzed CCR2 expression on these subsets and observed that its expression remains low and is not affected by infection (Fig 1H and 1I). These findings suggest that the regulatory effect of CCR2 on Th1, Th2, and Th17 cells may occur through indirect mechanisms. Considering the established role of CCR2 in promoting monocyte migration [9], we speculate that CCR2 influences T cell responses by regulating innate immune cells. This hypothesis is supported by our data showing upregulated CCR2 expression on pulmonary dendritic cells, macrophages, and monocytes following infection (Fig 1D and 1E), along with reduced immune cell infiltration observed in *ccr2*^-/-^ mice (Fig 3A–3D). Our ongoing research indicates that Ly6C^+^ pulmonary monocytes exhibit higher expression of F4/80 compared to CD11c. Co-culture experiments involving BMDMs derived from naïve *ccr2*^-/-^ or WT mice and spleen CD4^+^ T cells from naïve WT mice demonstrated a significant induction of IL-4 (Th2) rather than IFN-γ (Th1). These findings suggest that CCR2 may regulate macrophage function to influence T cell responses. Although these results provide valuable insights, they remain preliminary, and the detailed mechanisms underlying CCR2’s role in T cell regulation require further investigation.

CCR2 deficiency significantly impairs the clearance of *C*. *muridarum*, as demonstrated by higher chlamydial loads and exaggerated inflammatory responses in *ccr2*^-/-^ mice during the mid-phase, accompanied by increased weight loss and severe lung inflammation. These findings underscore the critical role of CCR2 in maintaining immune homeostasis and controlling pathological inflammation. Interestingly, despite their heightened susceptibility during the mid-phase, *ccr2*^-/-^ mice exhibit faster recovery in the late phase compared to WT mice. We speculate that this may be due to the activation of compensatory anti-inflammatory feedback mechanisms. These include the reduction of neutrophil infiltration (as shown in Fig 2F–2G, on day 14 p.i.), clearance of apoptotic neutrophils, the release of anti-inflammatory cytokines such as IL-10 and TGF-β [30], and the transformation of macrophages from classically to alternatively activated cells [31]. Our ongoing research supports this interpretation. Compared to WT mice, *ccr2*^-/-^ mice exhibit a reduction in pro-inflammatory, classically activated macrophages (CD80^+^, CD86^+^, MHC II^+^, iNOS^+^F4/80^+^, TNFα^+^F4/80^+^) and an increase in anti-inflammatory, alternatively activated macrophages (CD206^+^, ARG-1^+^F4/80^+^, IL-10^+^F4/80^+^, TGFβ^+^F4/80^+^) in the lungs following *C*. *muridarum* infection. Collectively, these mechanisms may reduce lung inflammation and help restore tissue homeostasis while also limiting Chlamydia growth. This compensatory response could explain the similar body weight and IFU levels observed in both mouse models. It highlights the importance of a balanced immune response in Chlamydia infections, where controlling the pathogen without causing excessive tissue damage is crucial.

Naïve CD4^+^ T cells are induced to differentiate into mature effector T cells (Th1, Th2, and Th17 cells) and regulatory T (Treg) cells. The expression of CCR2 is known to be inducible and increases in inflammatory lesions, thereby playing a critical role in recruiting and differentiating effector T cells. In *M*. *tuberculosis* infection, *ccr2*^-/-^ mice exhibit delayed T cells priming in MLNs, fewer IFN-γ-secreting CD4^+^ and CD8^+^ T cells in the lung, and a rapidly progressive course of disease [12]. In *Leishmania major* infection, CCR2 deficiency disrupts DCs migration to draining lymph nodes and alters splenic DCs composition, leading to impaired cell-mediated immune responses and a Th2 cytokine-biased nonhealing phenotype [32]. In this study, we observed a significant reduction in the infiltration of myeloid cells (including Mo, DC, and Mφ) and T cells in the lungs of *ccr2*^-/-^ mice (Fig 3). Notably, CD3^+^ T cells were significantly reduced on days 3 and 14 p.i., primarily due to a marked decrease in CD8^+^ T cells, while CD4^+^ T cell numbers remained comparable to those in WT mice. Given the critical role of CD8^+^ T cells in immune protection and immunopathology through their recognition of Chlamydia-infected cells [26], their reduced numbers likely contributed to the impaired bacterial control in *ccr2*^-/-^ mice. Furthermore, we observed a significant decrease in the activity of CD3^+^, CD4^+^, and CD8^+^ T cells in the lungs. We speculate that this reduced activity may result from diminished recruitment of lung antigen-presenting cells to interact with T cells in the absence of CCR2. In conclusion, these results demonstrate that CCR2 is crucial for coordinating myeloid and T cell responses during *C*. *muridarum* infection, highlighting its role in bacterial clearance and regulation of immunopathology.

Diminished T cell activity notably impairs the differentiation of Th1 cells and the secretion of IFN-γ. Th1 cells are critical for the clearance of intracellular pathogens, including Chlamydia, due to their ability to produce pro-inflammatory cytokines and promote Mφ activation. The observed impairment in Th1 cell differentiation and function in *ccr2*^-/-^ mice is concerning, as it reflects a disruption in the cytokine milieu necessary for promoting effective T cell responses. This disruption is further evidenced by the reduced expression of key Th1-promoting cytokines and transcription factors, such as *IFN-γ*, *il-12* and *T-bet*, highlighting the detrimental impact of *ccr2* deficiency on the development of robust Th1 responses. Importantly, *ccr2* deficiency also markedly decreased IFN-γ-secreting CD8^+^ T cells in the lung. This impairment of IFN-γ production disrupts the overall immune response to *C*. *muridarum*, leading to prolonged infection and exacerbated inflammation.

Conversely, our findings reveal that CCR2 deficiency leads to excessive Th2-type immune responses, characterized by increased production of IL-4 and IL-5, which compromise the host’s ability to effectively combat intracellular Chlamydia infections. *Ccr2*^-/-^ mice exhibited exaggerated Th2 cell responses in the lung and spleen following infection. IL-4, a key cytokine for Th2 cells, showed significantly increased mRNA expression on day 7 p.i. and protein levels on day 14 p.i. in the lungs of *ccr2*^-/-^ mice. STAT6, a critical transcription factor for Th2 cells, promotes Th2 differentiation and function via IL-4 signaling by upregulating GATA3 expression [33,34]. In this study, we observed significantly increased STAT6 expression and phosphorylation in the lung tissues of *ccr2*^-/-^ mice. Consequently, Gata3 expression was markedly upregulated under these conditions of elevated IL-4. Paradoxically, despite elevated IL-4 levels on day 14 p.i., *ccr2*^-/-^ mice showed comparable body weight and IFU to WT mice. IL-4 is recognized for its anti-inflammatory properties, modulating T cell differentiation and macrophage polarization to maintain tissue homeostasis [35]. We speculate that the elevated IL-4 in *ccr2*^-/-^ mice may reflect an anti-inflammatory feedback mechanisms, although further studies are needed to confirm this hypothesis. Additionally, we observed significant upregulation of IL-5 mRNA in the lungs of *ccr2*^-/-^ mice on day 3 p.i., which coincided with severe pulmonary inflammation and increased Chlamydia burden. This suggests that IL-5 may contribute to Th2-mediated immunopathology during *C*. *muridarum* infection in *ccr2*^-/-^ mice. Future research will focus on elucidating this mechanism. In conclusion, CCR2 plays a critical role in preventing Th2-mediated tissue damage, maintaining immune homeostasis, and reducing lung inflammation during *C*. *muridarum* infection.

Several studies have demonstrated a correlation between CCR2 and Th17 cells, revealing that mice lacking CCR2 exhibit exacerbated collagen-induced arthritis due to increased Th17 cell activity and elevated levels of IL-17A, IL-6, and IL-1β [36]. During the chronic phase of EAE, CCR2 enhances the inflammatory potential of developing Th17 cells, revealing a novel, time-dependent recruitment mechanism that amplifies T-cell-driven inflammation [22]. In this study, we observed elevated IL-17 levels and an enhanced Th17 immune response in the lung of *C*. *muridarum*-infected *ccr2*^-/-^ mice. The upregulation of STAT3 and its downstream target RORγt promotes Th17 differentiation and increased IL-17 production. IL-17/Th17 promotes type 1 T cell immunity against pulmonary intracellular bacterial infection through modulating DC function [25]. However, excessive IL-17 produced by Th17 cells promotes inflammatory responses and mediates neutrophil recruitment to the lung, leading to exacerbated lung inflammation during *C*. *muridarum* respiratory infection [20,37]. Indeed, in Result 2 of this study, we observed an enhanced recruitment of neutrophils in the lung of *ccr2*^-/-^ mice, accompanied by more severe lung inflammation and tissue damage. This finding suggests that the absence of CCR2 may facilitate excessive IL-17 secretion, thereby driving the over-recruitment of neutrophils and subsequently exacerbating pulmonary inflammation and tissue damage.

Foxp3^+^ regulatory T cells cells have an immunosuppressive function and antagonize Th17 cell-mediated immune responses [38]. Interestingly, while CCR2 deficiency amplified Th17-type responses, it did not impact the frequencies of Treg cells. The observation that *ccr2*^-/-^ mice exhibit comparable Treg response to WT mice during *C*. *muridarum* respiratory infection highlights the intricate and redundancy of immune regulatory mechanisms, suggesting that CCR2 may not be essential for Treg cells function and alternative pathways might influence it.

## Conclusion

Our study emphasizes the multifaceted role of CCR2 in modulating immune responses during *C*. *muridarum* respiratory infection. CCR2 is crucial for recruiting innate immune cells and plays a significant role in the differentiation and activation of adaptive immune cells, particularly Th1, Th2 and Th17 cells. *C*. *muridarum* infections induce CCR2 responses that are associated with the development of Th1 responses, the secretion of IFN-γ, and the clearance of local infection. CCR2 deficiency results in a dysregulated immune response characterized by excessive Th2 and Th17 polarization, ultimately compromising the host’s ability to control the infection. These findings suggest that targeting CCR2 or its signaling pathways may offer therapeutic potential for enhancing immune responses and improving outcomes in Chlamydia infections. Future studies should explore the underlying mechanisms of CCR2-mediated immune modulation and its potential as a therapeutic target in managing *C*. *muridarum* and other intracellular bacterial infections.

## Supporting information

S1 Images The raw images used to support the findings of this study. (PDF)

S1 Data The raw data used to support the findings of this study. (XLSX)
