# Supplementary material for: CCR2 signaling regulates anti-chlamydia T cell immune responses in the airway
Source: PLoS Pathog. 2025 Feb 4;21(2):e1012912. doi: 10.1371/journal.ppat.1012912 (PMC11793788; doi:10.1371/journal.ppat.1012912)

Manuscript Figure 2H

WT-Ly6G^{+} original image

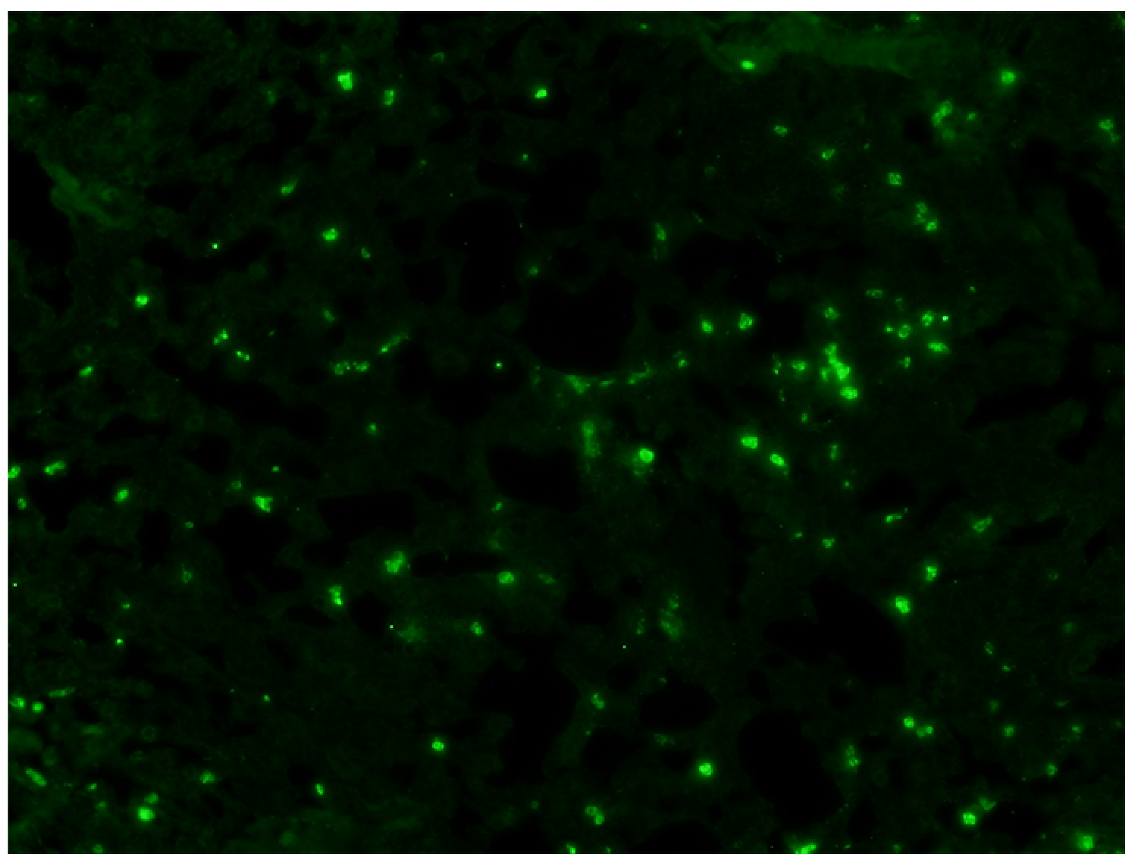

WT-DAPI original image

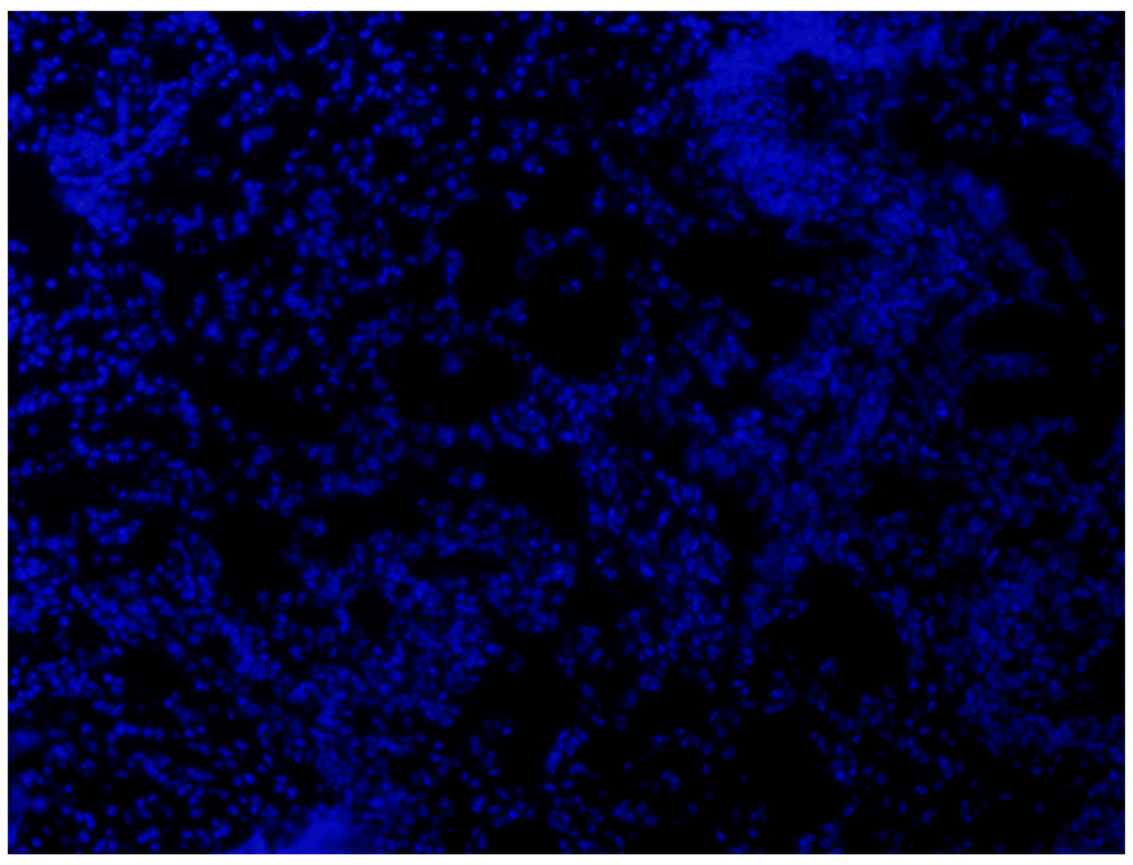

WT-Merge original image

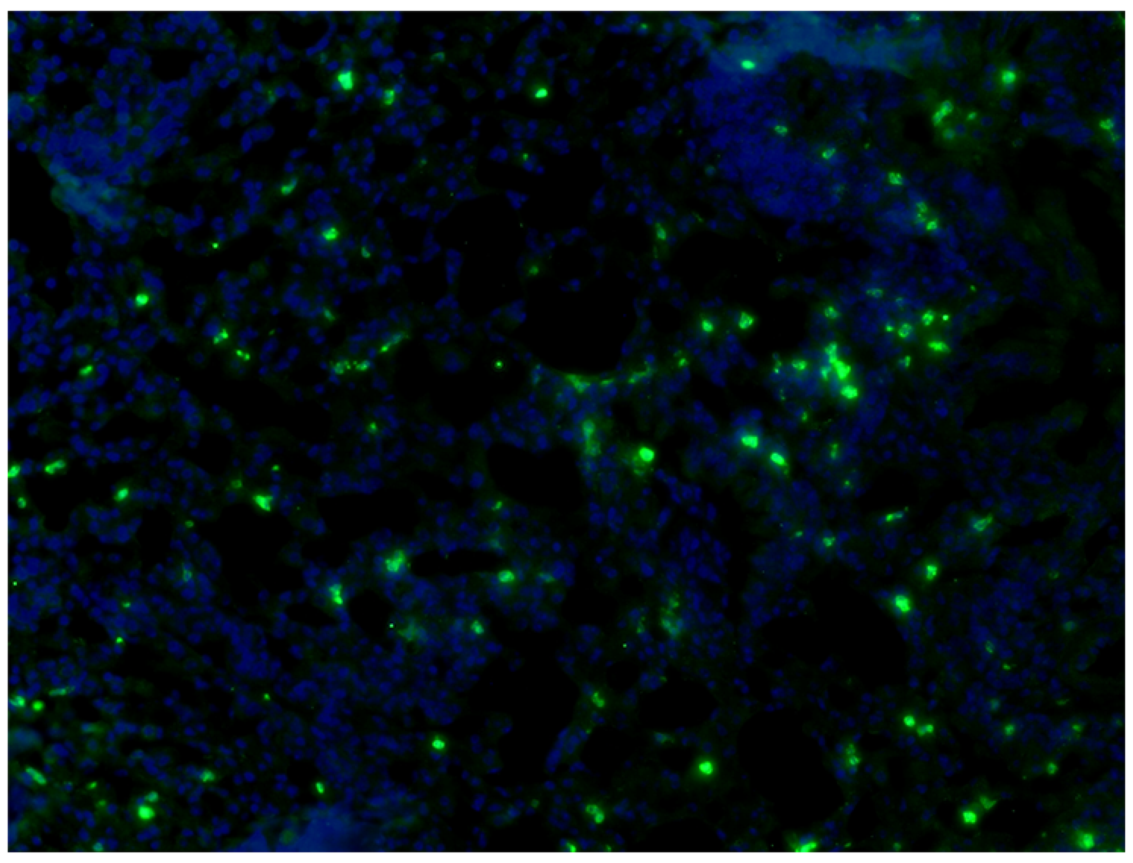

CCR2$^{-/-}$-Ly6G^{+} original image

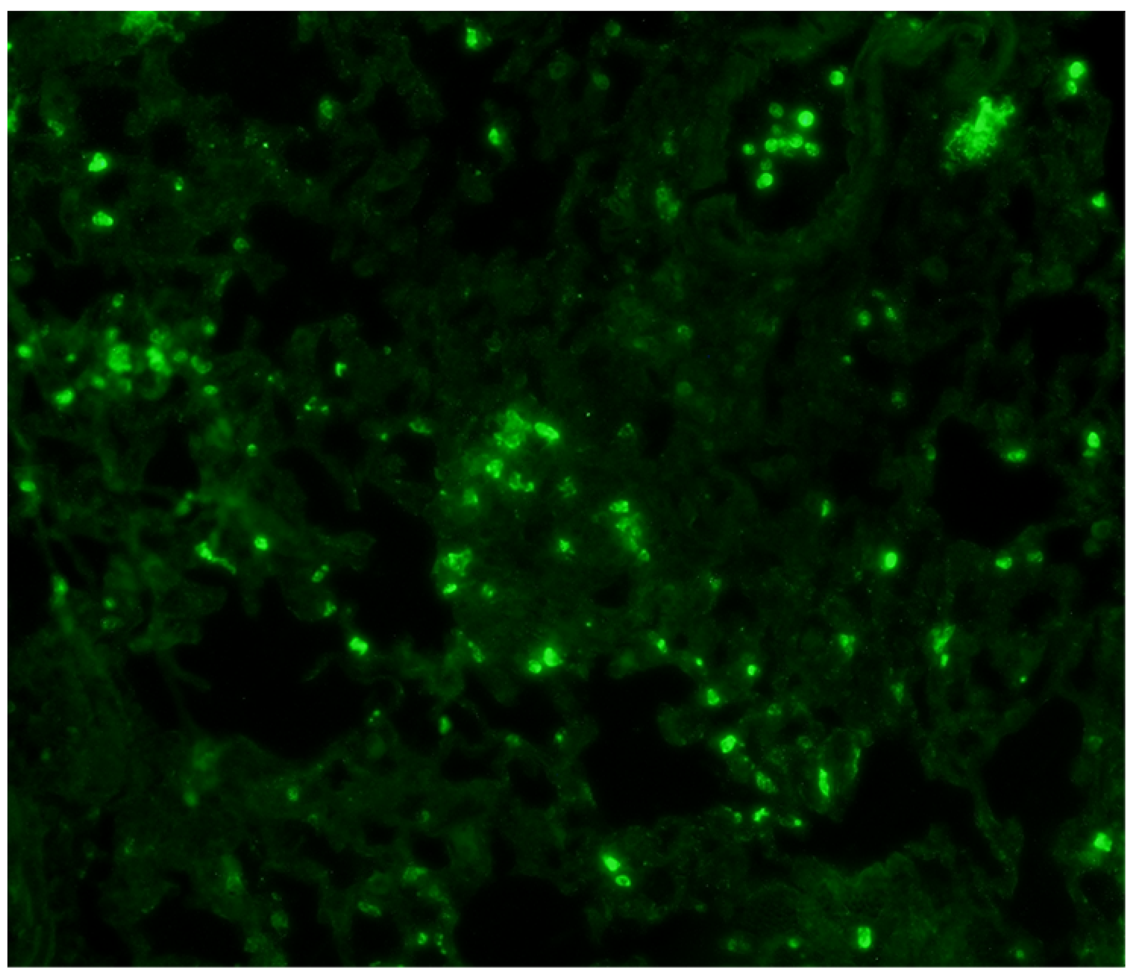

CCR2$^{-/-}$-DAPI original image

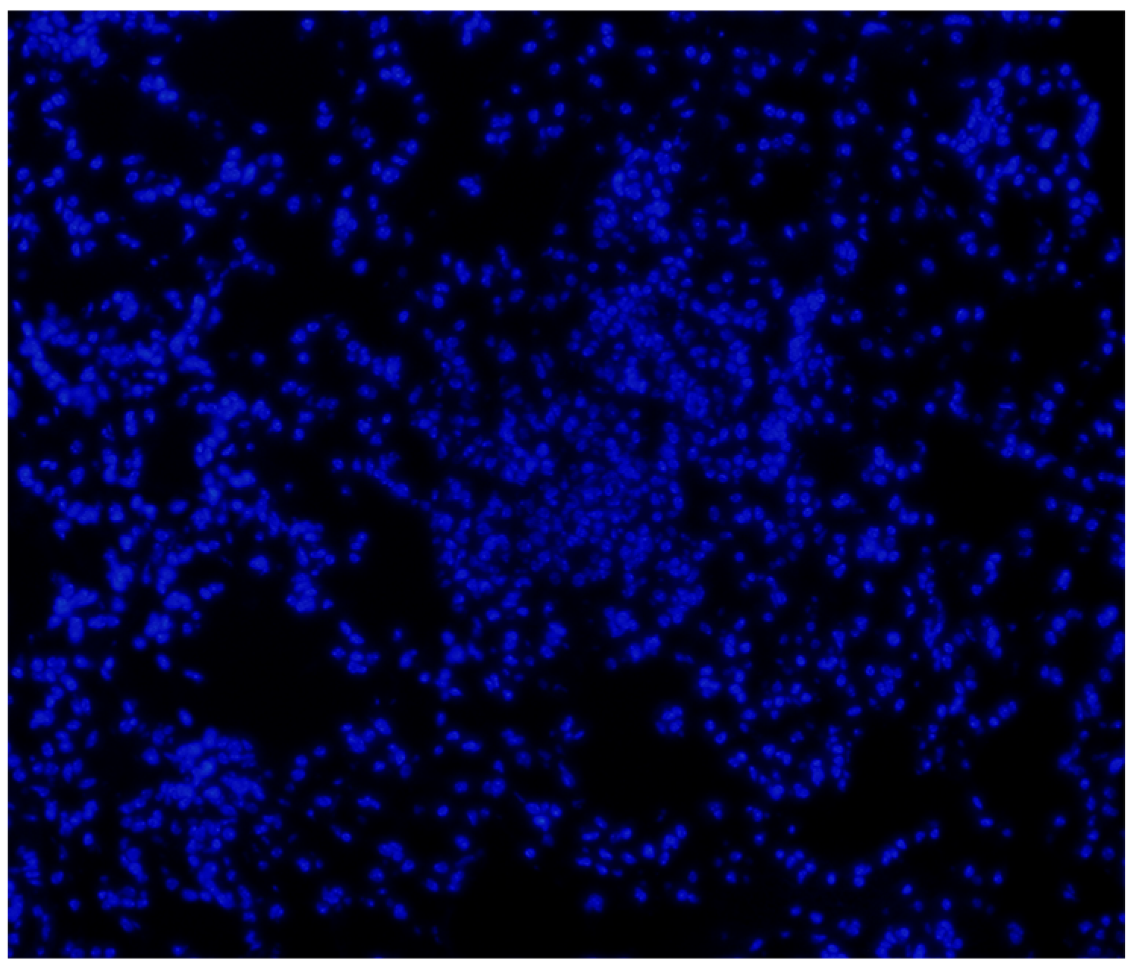

CCR2$^{-/-}$-Merge original image

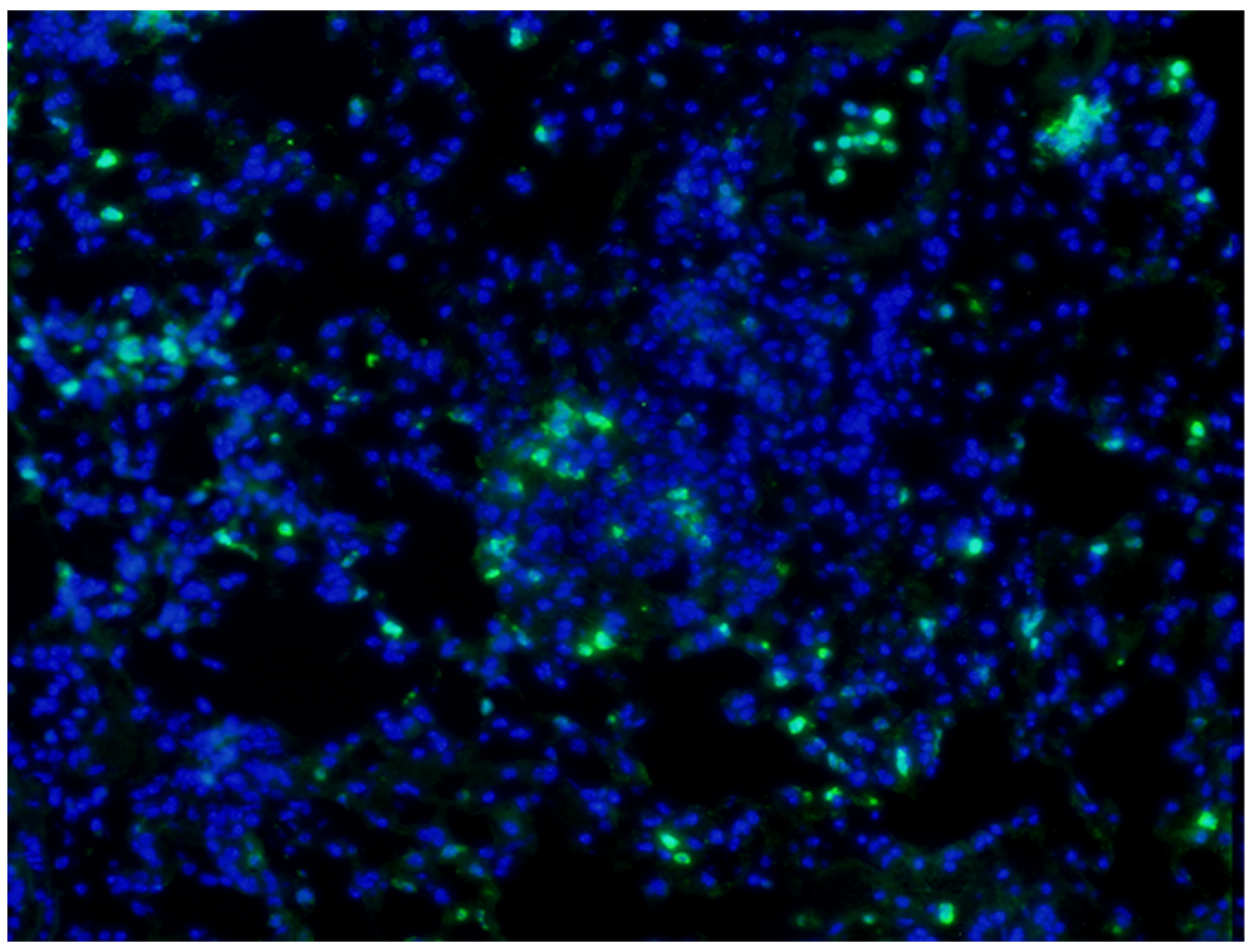

Manuscript Figure 4F

Original image | Manuscript image

STAT1

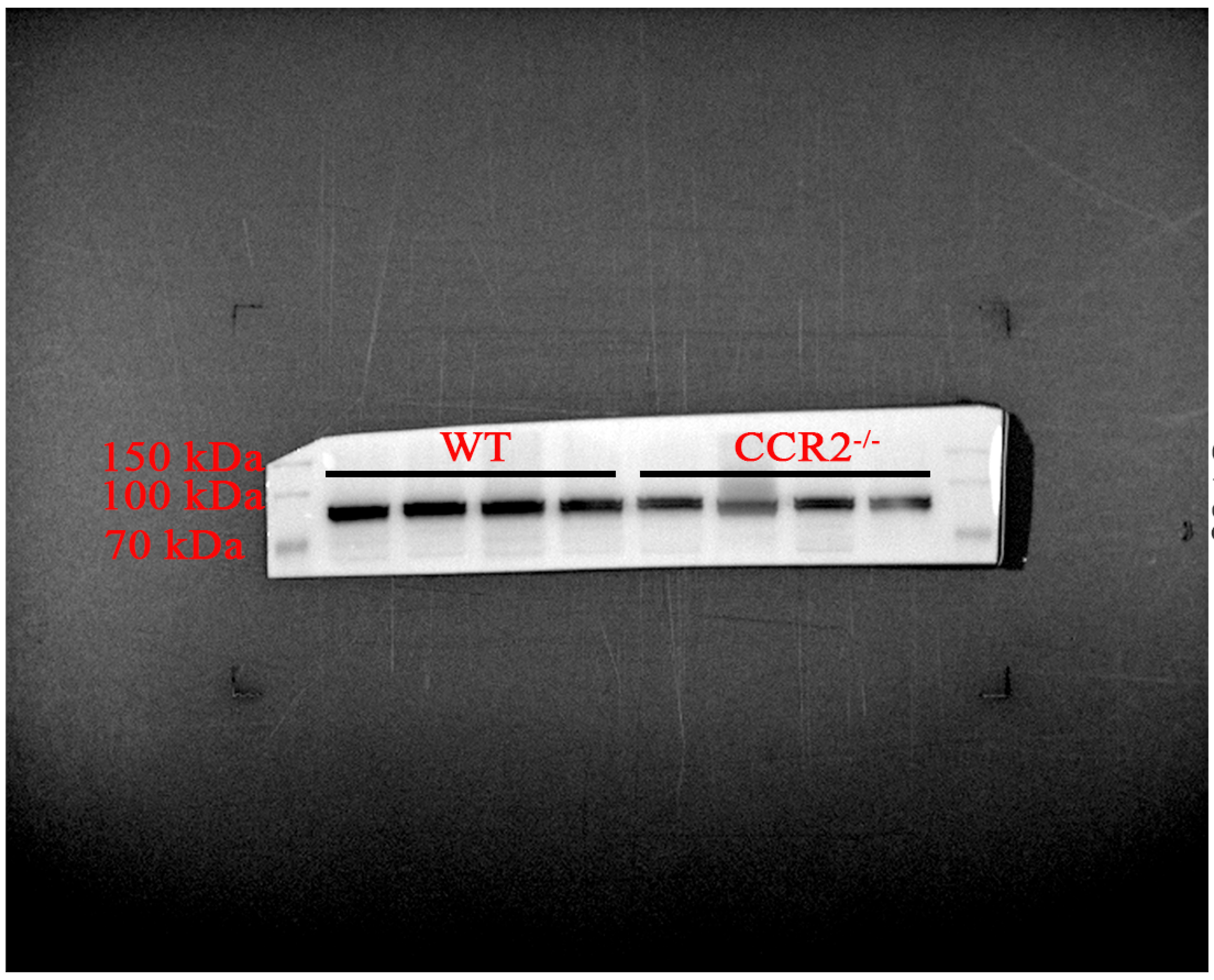

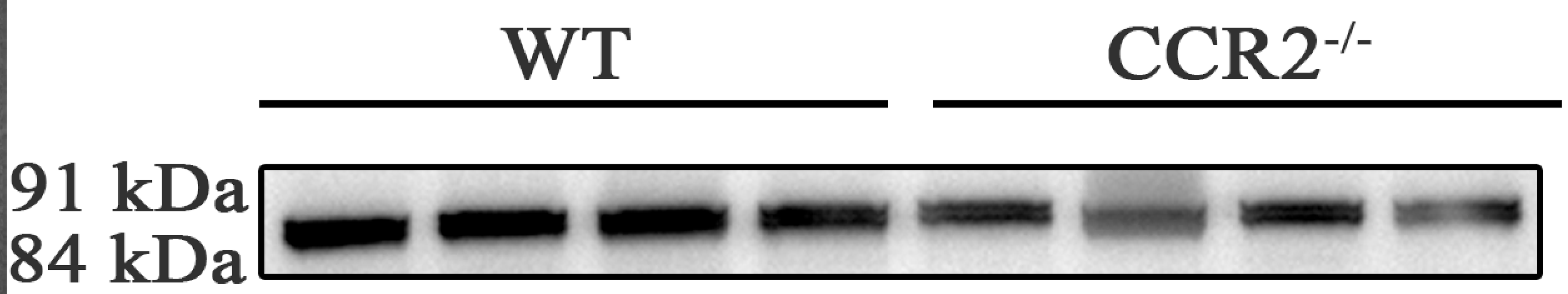

p-STAT1

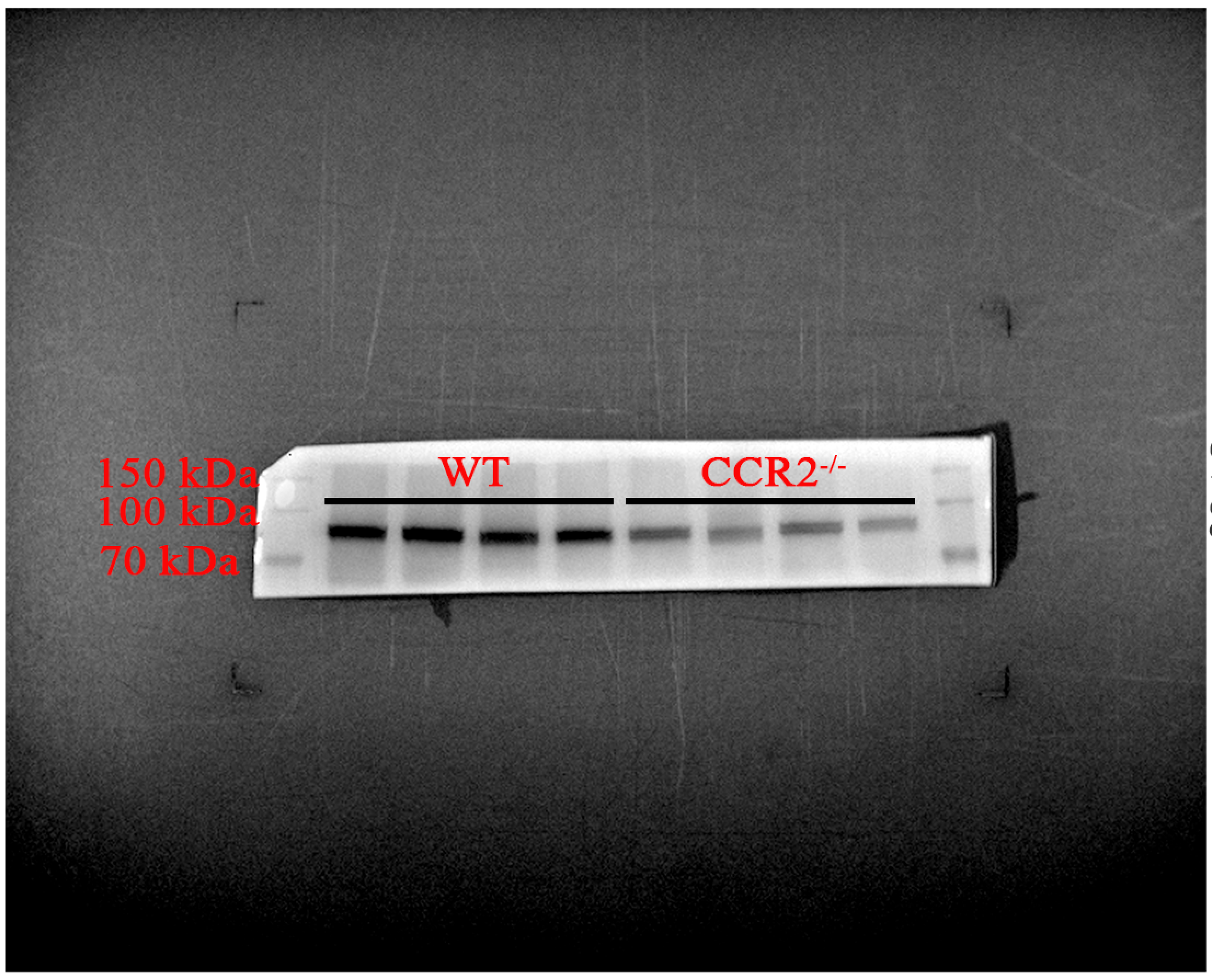

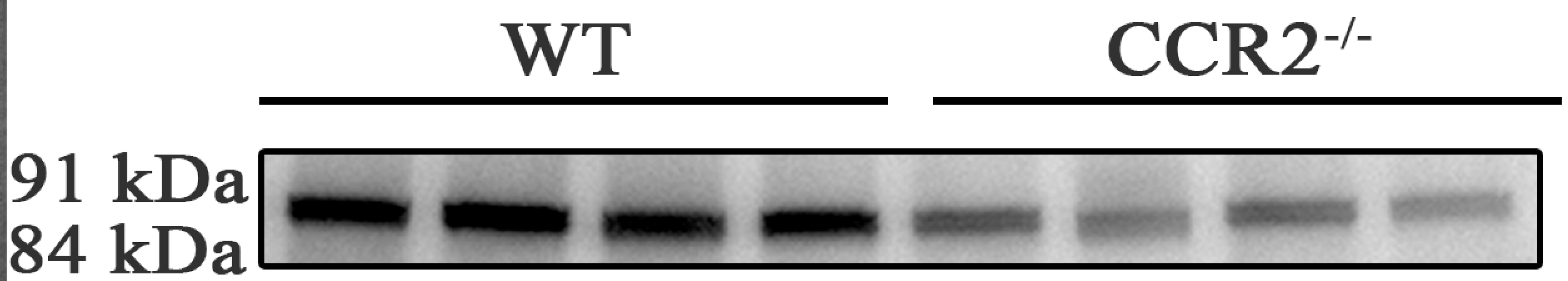

β-Actin

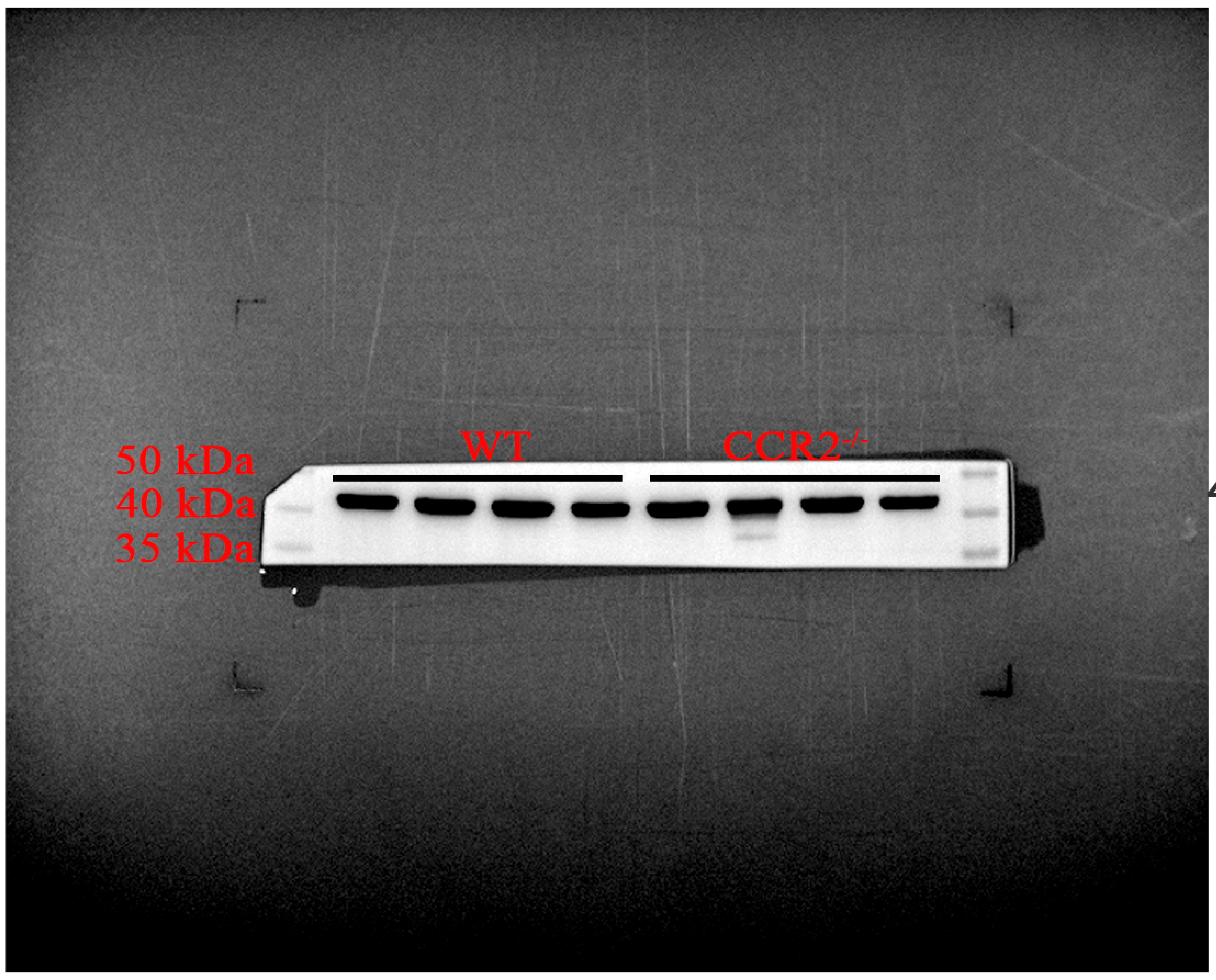

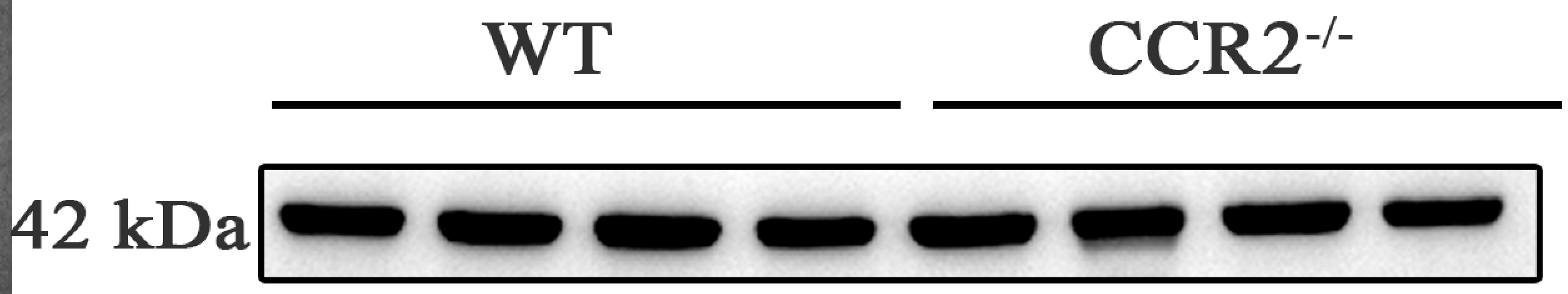

Manuscript Figure 5F

Original image

Manuscript image

STAT6

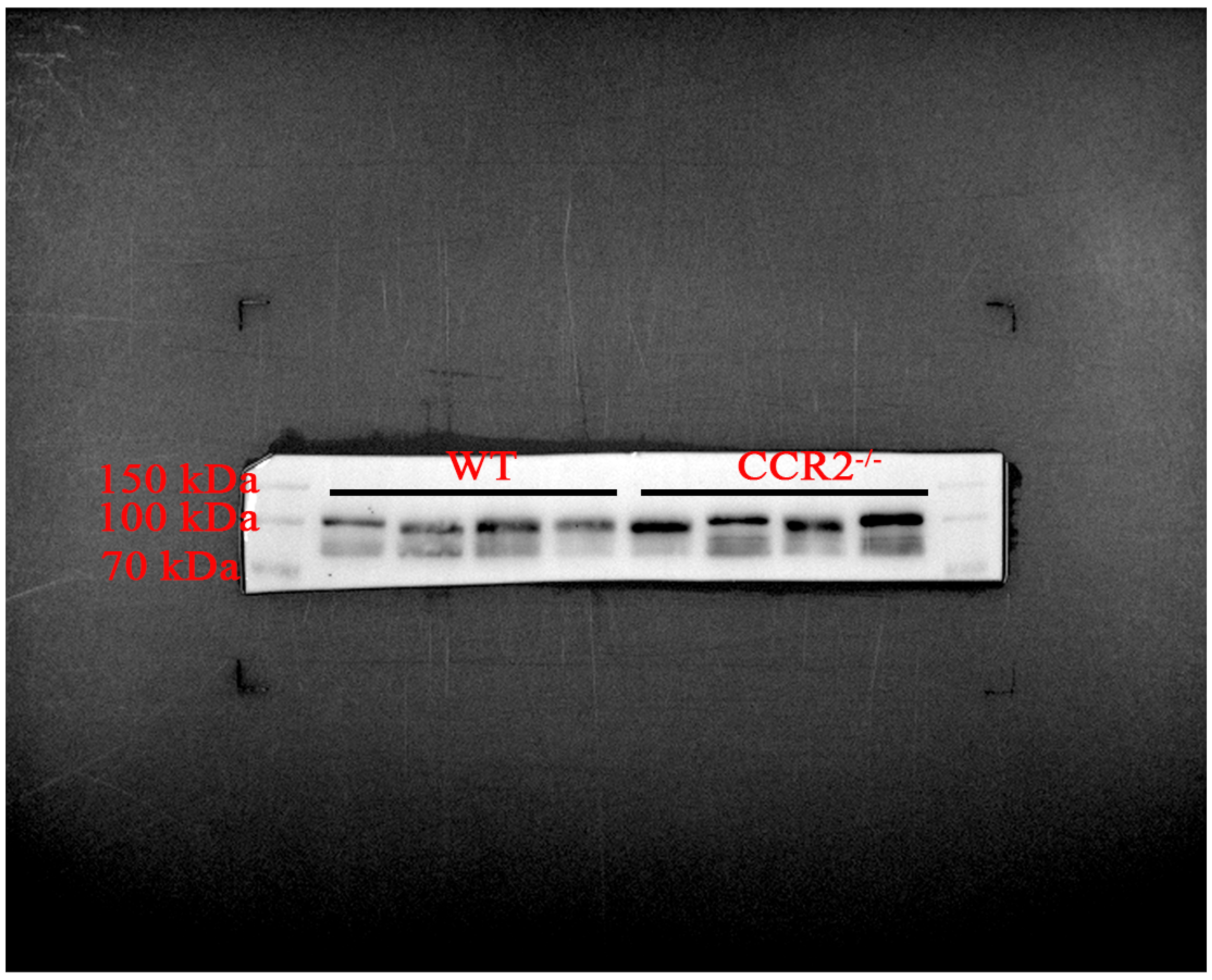

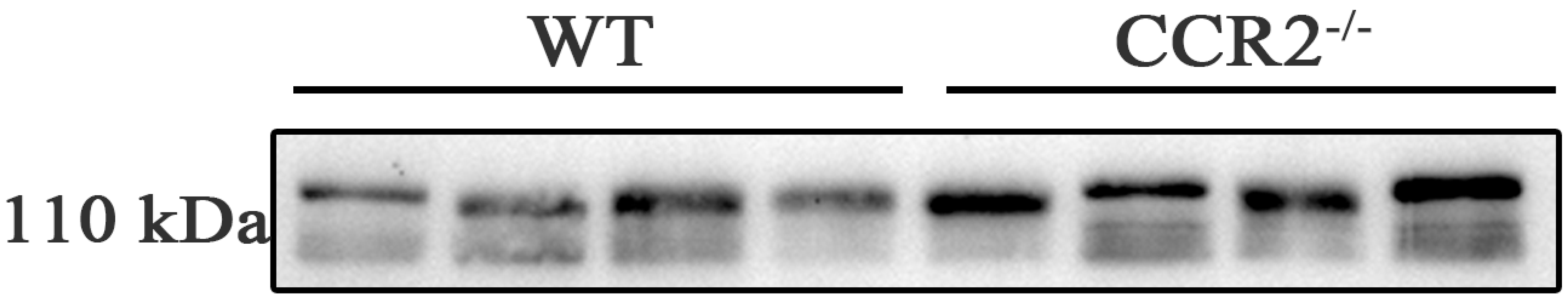

p-STAT6

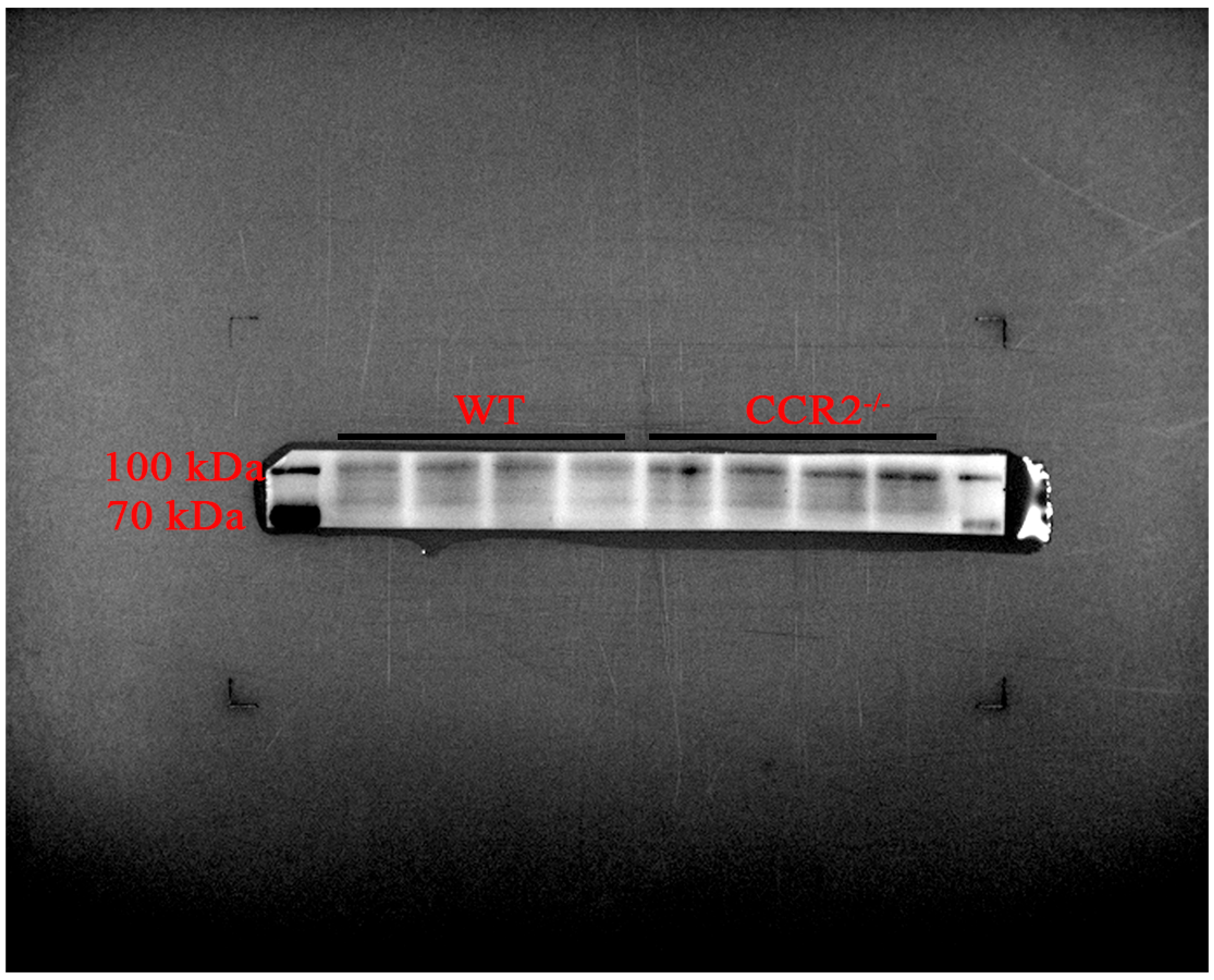

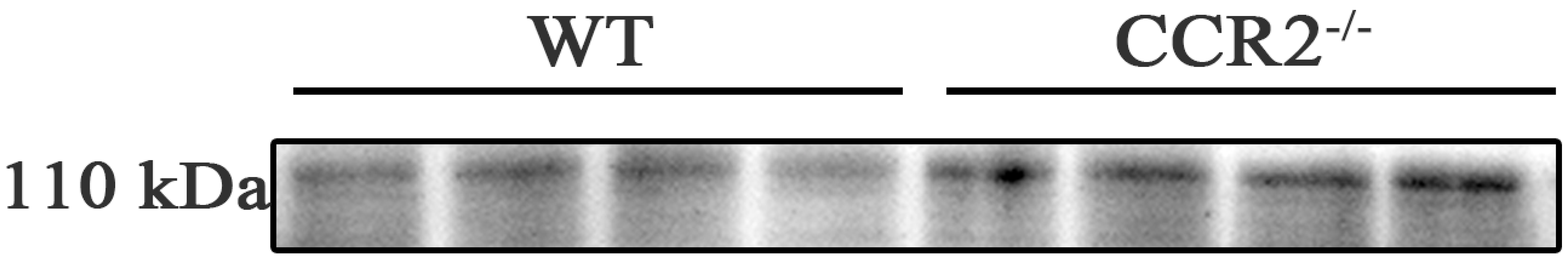

β-Actin

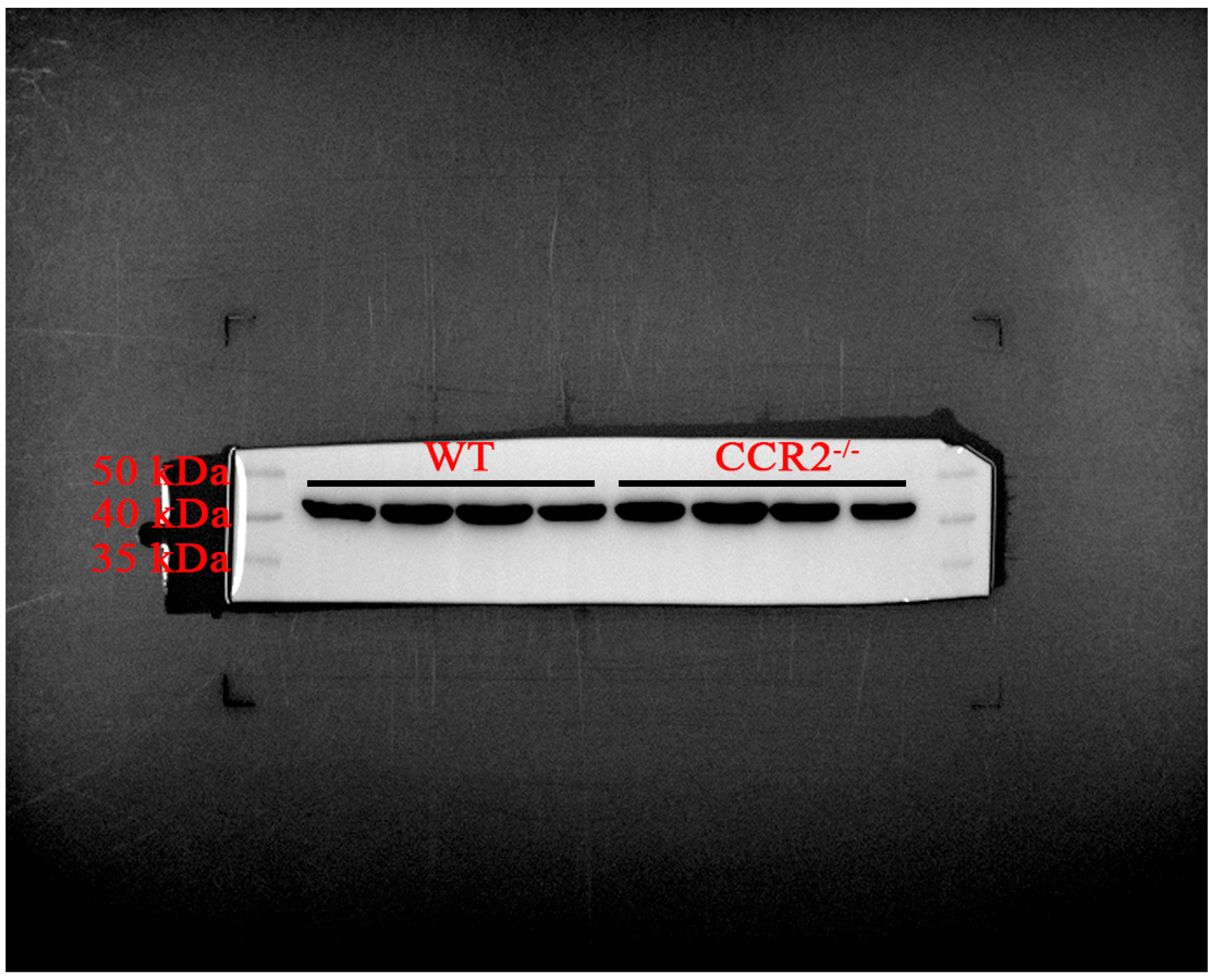

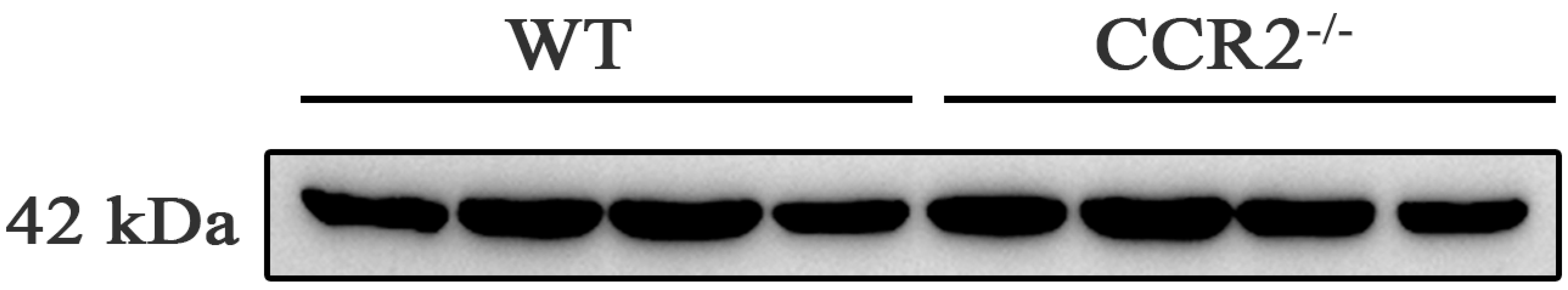

Supplement: S1 Images — (PDF) [file ppat.1012912.s001.pdf]
